# Network Pharmacology-Based Strategy to Investigate Pharmacological Mechanisms of the Drug Pair Astragalus-Angelica for Treatment of Male Infertility

**DOI:** 10.1155/2021/8281506

**Published:** 2021-10-16

**Authors:** Feng Zhao, Yingjun Deng, Guanchao Du, Shengjing Liu, Jun Guo, Hao Wang, Yazhou Chen, Fu Wang, Qiang Geng

**Affiliations:** ^1^Department of Andrology, Xiyuan Hospital of China Academy of Chinese Medical Sciences, No. 1 Xiyuan Playground, Zhongzhi Road, Beijing 100091, China; ^2^Department of Andrology, The First Teaching Hospital of Tianjin University of Traditional Chinese Medicine, No. 314 Anshan West Road, Tianjin 300193, China

## Abstract

**Background:**

The traditional Chinese medicines *Astragalus* and *Angelica* are often combined to treat male infertility, but the specific therapeutic mechanism is not clear. Therefore, this study applies a network pharmacology approach to investigate the possible mechanism of action of the drug pair *Astragalus*-*Angelica* (PAA) in the treatment of male infertility.

**Methods:**

Relevant targets for PAA treatment of male infertility are obtained through databases. Protein-protein interactions (PPIs) are constructed through STRING database and screen core targets, and an enrichment analysis is conducted through the Metascape platform. Finally, molecular docking experiments were carried out to evaluate the affinity between the target protein and the ligand of PAA.

**Results:**

The active ingredients of 112 PAA, 980 corresponding targets, and 374 effective targets of PAA for the treatment of male infertility were obtained, which are related to PI3K-Akt signaling pathway, HIF-1 signaling pathway, AGE-RAGE signaling pathway, IL-17 signaling pathway, and thyroid hormone signaling pathway.

**Conclusion:**

In this study, using a network pharmacology method, we preliminarily analyzed the effective components and action targets of the PAA. We also explored the possible mechanism of action of PAA in treating male infertility. They also lay a foundation for expanding the clinical application of PAA and provide new ideas and directions for further research on the mechanisms of action of the PAA and its components for male infertility treatment.

## 1. Background

Male sterility, a common disease in andrology, is defined as male factor infertility in which a couple have attempted to conceive for more than one year without success. Approximately 15% of couples do not achieve pregnancy within one year and seek medical treatment for infertility. One in eight couples encounters problems when attempting to conceive a first child, and one in six encounters problems when attempting to conceive a subsequent child. Three percent of women who are currently trying to conceive remain involuntarily childless, while 6% of parous women are not able to have as many children as they would wish [1]. In 50% of involuntarily childless couples, a male infertility-associated factor is found, usually together with abnormal semen parameters [2]. The etiology of male infertility is complex. At present, the clear etiologies include urogenital diseases, varicoceles, endocrine disorders, gene abnormalities, and other systemic diseases. In addition, the European Association of Urology Guidelines (2020 Edition) states that approximately 30% of infertility cases are idiopathic male infertility [3]. The existing treatment measures include estrogen receptor modulators, sperm health-promoting agents, antibiotics, and other symptomatic treatments, but the efficacies are still unclear [4, 5].

The treatment of male infertility with traditional Chinese medicine (TCM) has a long history and is now gaining popularity in western countries [6, 7]. The goal of treatment is the balance between reproductive energy (QI), blood, and visceral Yin and Yang. Usually, traditional Chinese medicine is not used alone to treat diseases, but often in the form of multiple drugs cooperating with each other. *Astragalus* and *Angelica* are often used as a drug combination in the treatment of male infertility. *Astragalus* is the dried root of *Astragalus membranaceus* (Fisch.) Bge. or *Astragalus membranaceus* (Fisch.) Bge. var. *mongholicus* (Bge.) Hsiao. [8]. The efficacy is tonifying qi and strengthening the spleen. *Angelica* is the root of *Angelica sinensis* (Oliv.) [9]. The efficacy is tonifying the blood and activating blood circulation. The combination of the two can treat male sterility with deficiency of qi and blood [10, 11]. However, it is not clear what mechanism PAA is involved in the treatment of male infertility.

**Figure 1:**
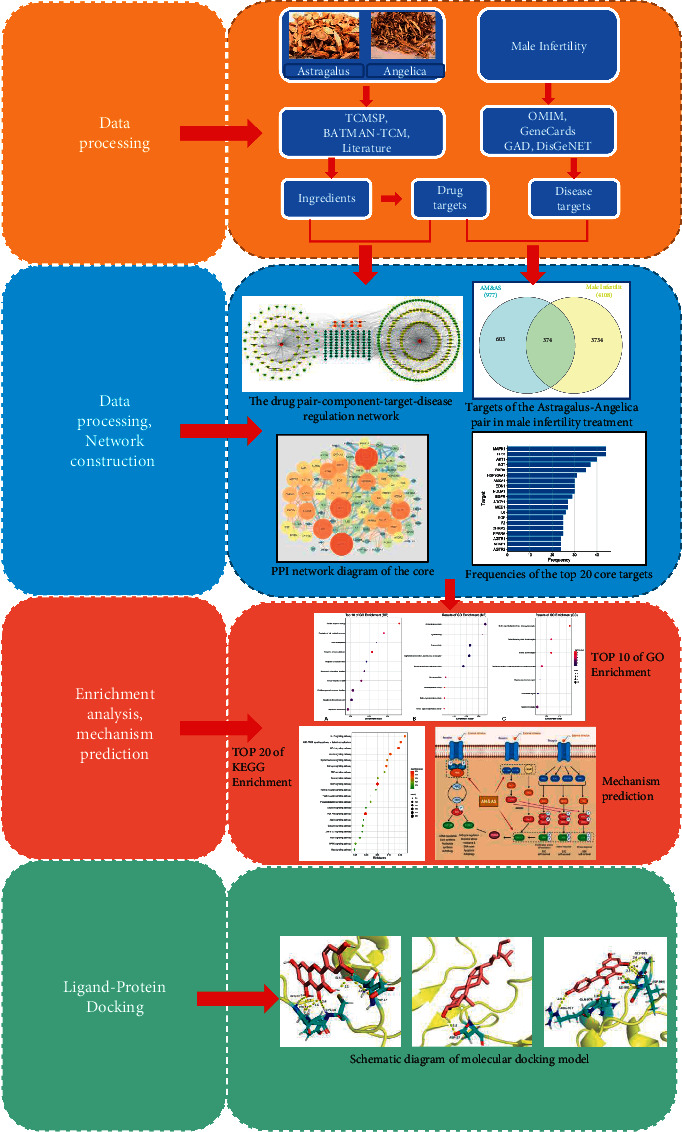
Technical strategy flow diagram.

Network pharmacology is a new discipline that has been used in recent years to study the pharmacological mechanisms of traditional Chinese medicines. Through integration of current traditional Chinese medicine pharmacology methods, high-throughput bioinformatics, and high-end data analysis software programs, comprehensive analyses of the mechanisms of action of traditional Chinese medicines can be carried out [12]. It can predict the pharmacological mechanism of TCM “multicomponent, multitarget, and multipathway,” and provide better data and theoretical support for subsequent pharmacological experiments. The current study is comprised of three steps, including data collection, data processing, and network construction, enrichment analysis and mechanism prediction, and the flowchart of the technical strategy in this study is shown in Figure 1.

## 2. Materials and Methods

### 2.1. Identification of Active Components and Potential Targets of the Traditional Chinese Medicines

The Traditional Chinese Medicine Systems Pharmacology Database and Analysis Platform (TCMSP) [13] is a relatively classic database of traditional Chinese medicine ingredients with more than 500 traditional Chinese medicines and more than 30,000 compounds providing pharmacokinetic information corresponding to the compounds. It has become one of the databases most frequently used by scholars in network pharmacology research. The Bioinformatics Analysis Tool for Molecular Mechanism of Traditional Chinese Medicine (BATMAN-TCM) [14] was the first online bioinformatics analysis tool designed specifically for studying the intrinsic molecular mechanisms of traditional Chinese medicines. Relevant active components of PAA were searched in the TCMSP and BATMAN-TCM databases with the following screening criteria, which were set in combination with the criteria of commonly used network pharmacology component screening methods and relevant literature records: an oral bioavailability (OB) ≥ 30% and drug likeness (DL) ≥ 0.18. Compounds that did not meet these screening criteria were excluded. And according to the literature, active ingredients that are not eligible for screening and that have been experimentally proved to be effective are also incorporated into the alternative ingredients; thus, the obtained active ingredients and therapeutic targets of *Astragalus and Angelica* were typed into the UniProt database for each target https://www.uniprot.org/ to obtain the standard number of targets, and the final results were counted into an Excel sheet.

### 2.2. Identification of Disease Targets

Online Mendelian Inheritance in Man (OMIM) (https://omim.org/) is a database of human genes and genetic phenotypes which focuses on molecular relationships between gene variation and dominant expression and contains information on more than 15,000 genes. GeneCards [15] (https://www.genecards.org/) contains a wealth of biomedical data on genes and their products, including genomic, proteomic, and gene function-related information. DisGeNET [16] is a multifunctional platform that can be used for the study of the molecular basis of human diseases and their complications, validation of disease candidate genes, and evaluation of the performance of text mining methods. The Genetic Association Database (GAD) [17] is a standardized tool for viewing the ever-growing data on human polymorphisms from case-control studies. Users can search for genes, diseases, polymorphisms, chromosome locations, and references to obtain relevant information. We input “male infertility” into the OMIM database, the GeneCards database, GAD (https://geneticassociationdb.nih.gov/), and DisGeNET (http://www.disgenet.org) to search for the targets related to male infertility and record them in an Excel sheet.

### 2.3. Identification of Therapeutic Targets and Construction of Protein-Protein Interactions (PPIs)

The effective components, targets, and disease targets of the PAA were deduplicated and imported into a Venn diagram program (http://bioinformatics.psb.ugent.be/web-tools/Venn/). Finally, the targets at the intersection were considered the effective targets of PAA in the treatment of male infertility. The Search Tool for the Retrieval of Interacting Genes/Proteins (STRING) can be used to judge the confidence of PPIs through different evidence scoring systems so that we will know which proteins play a major role in the treatment of diseases. The targets obtained after intersection analysis were input into the STRING to construct a PPI network, and the results were imported into Cytoscape software (version 3.7.2) [18]. According to the network analysis tool and the Molecular Complex Detection (MCODE) [19] plug-in, topology analysis was carried out to obtain the comprehensive data for each node. According to the betweenness centrality (BC), closeness centrality (CC), degree, and MCODE Score, the core targets of PAA in the treatment of male infertility were identified, and a PPI network was drawn.

### 2.4. Network Construction

The disease-drug-active ingredient-target network of PAA in the treatment of male infertility was visualized by using Cytoscape software. In the network, each node represents a data element, such as a disease, drug, active ingredient, or target. In the scale-free network, the index node degree reflects the participation of each node, and the size of the degree is directly proportional to the participation of the node. After importing the data, the Network Analyzer module was used to obtain the degree of each data element.

### 2.5. Functional Nodes for Core Targets

Metascape (http://metascape.org/) is a powerful tool for gene function annotation analysis that can help users apply the current popular bioinformatics analysis methods to batch gene and protein analysis in order to understand gene or protein functions. In this study, we used the Metascape database to perform gene ontology (GO) functional annotation and Kyoto Encyclopedia of Genes and Genomes (KEGG) pathway enrichment. GO analysis was used to describe the functions of gene targets in terms of cellular component (CC), molecular function (MF), and biological process (BP). KEGG analysis was used to identify the signaling pathways associated with the gene targets, and the analysis results with statistical significance were selected (*P* < 0.05). A network of the interactions between the core targets and signaling pathway was drawn with Cytoscape software to obtain the comprehensive information for each node.

### 2.6. Construction of the Component-Target Pathway Network

The top 20 pathways from the results of KEGG analysis were filtered and combined with the file retrieval to obtain the possible pathways related to the treatment of male infertility and the targets that were enriched on the pathways. The targets connected with the effective components in the PAA pair were ultimately used to construct a component-target pathway network diagrams.

### 2.7. Ligand-Protein Docking

The information of six proteins (TP53, mapk1, IL-6, ANXA1, EGF, and EGFR) in the PPI network which were most closely related to male infertility was obtained through the RCSB PDB (https://www.rcsb.org/). The proteins selected as .pdb format were converted into .pdbqt format via AutoDock (http://autodock.scripps.edu/). Docking was performed in AutoDock with kaempferol, stigmasterol, and quercetin.

## 3. Results

### 3.1. Identification of Ingredients and Targets of the Traditional Chinese Medicine

The effective components of PAA were searched in the TCMSP and BATMAN-TCM, and the compounds were screened according to an OB ≥ 30% and a DL ≥ 0.18 and combined with the literature for screening [20–23]. Ultimately, 112 potential core compounds were obtained, and 977 corresponding targets were predicted.

**Figure 2:**
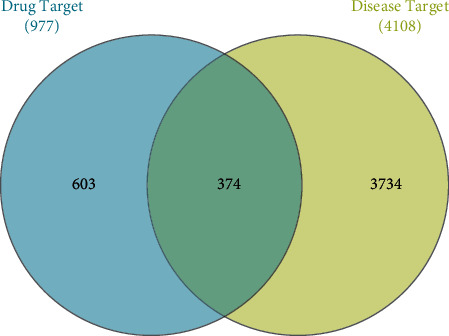
Targets of the PAA in male infertility treatment. The number in the blue circle is the unique target number of AS&AN, the number in the yellow circle is the unique target number of male infertility, and the middle number is the common target number common for both.

The OMIM database, GeneCards database, GAD, and DisGeNET database were used to search for male infertility-related targets. A total of 4108 targets were obtained after deleting the duplicate items. Finally, 374 effective targets for the treatment of male infertility were obtained by mapping (Figure 2).

### 3.2. Construction of the PPI Network

**Figure 3:**
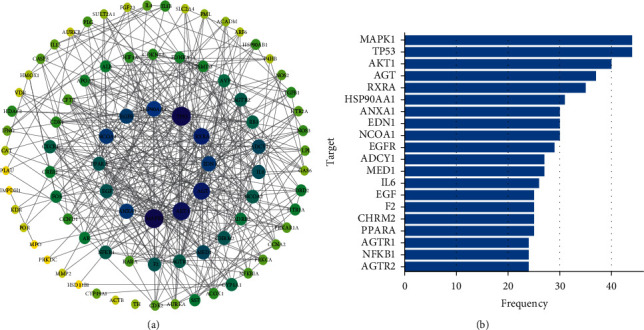
The PPI networks of AS&AN for male infertility. (a) PPI network diagram of the core. (b) Frequencies of the top 20 core targets (the *y*-axis represents targets, and the *x*-axis represents the frequency).

A total of 374 common targets were imported into the STRING for PPI analysis, and the result with a combined score ≥0.900 was selected. A total of 1415 paired interaction relationships were obtained. The results were imported into Cytoscape, and after screening was performed on the basis of betweenness centrality, closeness centrality, degree, and MCODE score, 85 core targets were obtained. The PPI network was drawn with these targets, as shown in Figure 3(a). A bar chart was also created to show the frequency of occurrence of the top 20 targets, as shown in Figure 3(b) and Table 1.

### 3.3. Construction of the Drug Pair-Component-Target-Disease Regulation Network

**Figure 4:**
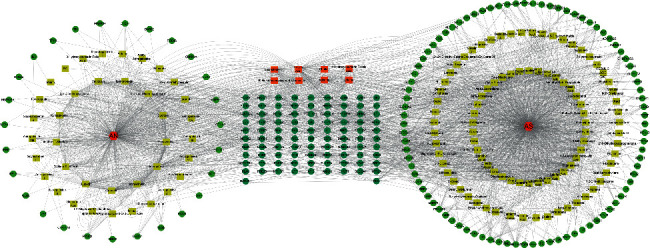
The drug pair-component-target-disease regulation network. The red node is the traditional Chinese medicine, the yellow node is the active ingredient, the green node is the target, the orange node between the two circles is the common active ingredient of *Astragalus* and *Angelica*, and the dark green node is the common target of *Astragalus* and *Angelica*.

The common target information and mapping relationships between the active components of the PAA pair and male infertility were imported into Cytoscape software. The topological structure of the network was assessed with the Network Analyzer function of Cytoscape, and the node importance was expressed in terms of the degree. Nodes with degrees greater than 20 for the active ingredients and 10 for the targets were selected, and a drug pair-component-target network diagram was established, as shown in Figure 4. The top five compounds with regard to degree were kaempferol, stigmasterol, beta sitosterol, quercetin, and canavanine. Among them, kaempferol, stigmasterol, and quercetin are closely related to male infertility.

### 3.4. GO Enrichment Analysis of Core Targets

**Figure 5:**
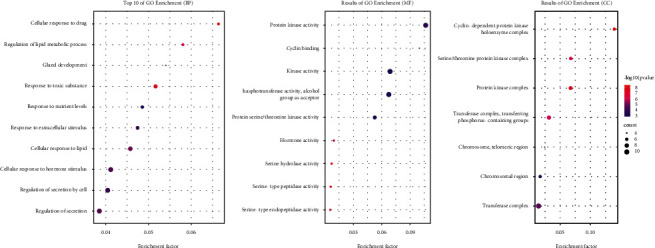
Top 10 of GO enrichment: (a) top 10 of GO enrichment (BP), (b) results of GO enrichment (MF), and (c) results of GO enrichment (CC) (the *y*-axis represents top 10 BP/MF/CC terms, and the *x*-axis represents the enrichment factors.).

GO target analysis yielded 398 significant results, including 382 BPs, 7 CCs, and 9 MFs. The top 10 BP, CC, and MF terms were used to create bubble charts, as shown in Figure 5. In the BP category, the main terms associated with the targets included the cellular response to drug, regulation of secretion, and response to nutrient levels terms. In the CC category, the enriched terms for the targets included the protein kinase complex, transferase complex, and chromosomal region terms. In the MF category, the targets were enriched for terms such as kinase activity, cyclin binding, and hormone activity.

### 3.5. KEGG Analysis of Core Targets

**Figure 6:**
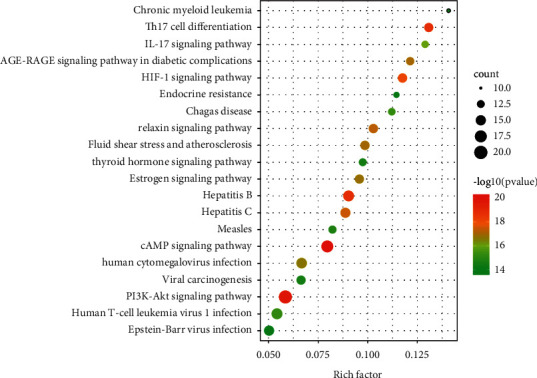
Top 20 of KEGG enrichment (the *y*-axis represents top 20 of KEGG terms, and the *x*-axis represents the rich factors).

A total of 153 pathways were obtained through KEGG enrichment analysis. Combined with literature search, the top 20 pathways with high significance were selected for bubble mapping based on the size of *P* value. A literature search revealed that among the 20 pathways were important signaling pathways related to infertility, including the PI3K-Akt, HIF-1, AGE-RAGE, IL-17, and thyroid hormone signaling pathways. These pathways all involved targets such as TP53, mapk1, IL-6, ANXA1, EGF, and EGFR, and detailed information is available in Table 2. These results are shown in Figure 6.

### 3.6. Results of Docking

**Figure 7:**
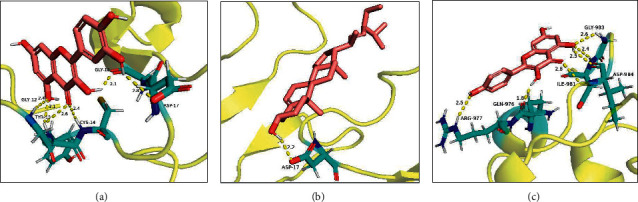
Schematic diagram of the molecular docking model. (a) Schematic diagram of docking between EGF and quercetin. (b) Schematic diagram of docking between EGF and stigmasterol. (c) Schematic diagram of docking between EGFR and kaempferol.

A total of 18 docking results were obtained, as shown in Table 3. The results of this study show that the binding energies of the ligands to the receptors are all negative, which indicates that there is binding activity between the compounds and the target protein, strong binding activity when the binding energy is less than −5 kJ/mol, and strong binding activity when it is less than −7 kJ/mol. The docking mode at which each ligand had the lowest binding energy was selected for display (see Figure 7).

## 4. Discussion

A previous report on the use of traditional Chinese medicines for the treatment of male infertility indicated that both *Astragalus* and *Angelica* are very frequently used in traditional Chinese medicines [24]. They are often used to treat infertility due to deficiency of both qi and blood. According to the theory of traditional Chinese medicine, Qi can generate blood, and blood can carry Qi. Qi deficiency cannot generate blood, and blood deficiency cannot transform essence. *Astragalus* and *Angelica* can supplement both qi and blood and regulate Yin and Yang.

A total of 112 effective components, 980 corresponding targets, and 4108 disease targets of the PAA pair were screened from the component database, and 374 potential targets for male infertility were obtained after mapping. This finding shows that PAA has multicomponent and multitarget characteristics in the treatment of male infertility. The drug pair-component-target network diagram shows that kaempferol, stigmasterol, beta sitosterol, quercetin, and canavanine are the top active ingredients. The association of these several components with male infertility is as follows. Kaempferol is a flavonoid that can significantly increase the levels of antioxidants such as SOD (superoxide dismutase), CAT (catalase), and GPX (glutathione peroxidase) in the sperm of diabetic rats; reduce the levels of inflammatory factors such as NF-*κ*B (nuclear factor kappa B) and TNF-*α* (tumor necrosis factor alpha) in sperm; alleviate sperm damage [25]; and significantly improve sperm quality in mice with infertility induced by benzopyrene, a product of incomplete combustion of energy substances [26]. Quercetin and other flavonoids also have good anti-inflammatory effects [27]. Relevant studies have shown that after treatment with quercetin, the content of mtDNA (mitochondrial DNA) in patients' sperm significantly decreases, while the content of Cyt b (cytochrome b) and NADH 5 (nicotinamide adenine dinucleotide 5) in sperm significantly increases, which plays a role in improving sperm hyperactivity and the acrosome reaction [28]. Stigmasterol belongs to the class of phytosterols. Studies have shown that dietary phytosterol supplementation can significantly increase the sperm number and sperm motility and reduce the sperm oxidative stress response [29]. Therefore, kaempferol, stigmasterol, quercetin, and other compounds may be the core compounds by which PAA alleviates male infertility.

The results showed that the potential targets of *Angelica* and *Astragalus* in the treatment of male infertility include TP53 (tumor protein P53), mapk1 (mitogen-activated protein kinase 1), IL-6 (interleukin-6 receptor), ANXA1 (annexin-A1), EGF (epidermal growth factor), and EGFR (epidermal growth factor receptor). Here, the relationship between these six targets and male infertility is described. The TP53 gene, an important tumor suppressor gene, can regulate cell growth, differentiation, aging, and the immune response. Meiosis is crucial in spermatogenesis, and TP53 mRNA and protein are expressed in primary spermatocytes and play important roles in spermatogenic cell apoptosis, suggesting that TP53 participates in meiosis [30, 31]. A growing number of studies on cancer have confirmed that the PI3K-Akt pathway can provide positive and negative regulation of p53 levels [32]. However, whether this regulation affects male fertility is not yet evidenced. MAPK (mitogen-activated protein kinase) is an important part of the MAPK signaling pathway. MAPK is closely related to proliferation, inflammation, differentiation, apoptosis, and other processes in male sperm [33]. ANXA1, an important member of the mammalian annexin family, is closely related to cell proliferation and membrane fusion [34]. Studies on human sperm have found that ANXA1 can bind and interact with F-actin in a Ca2+-dependent manner [35], and the actin cytoskeleton plays an important role in sperm capacitation and the acrosome reaction [36]. IL-6 is an inflammatory cytokine secreted by macrophages. Although it is mainly involved in the inflammatory reaction in vivo, its levels are significantly increased in the semen of infertile patients [37]. EGF is closely related to the reproductive activity of male animals. An appropriate dose of EGF can specifically promote the proliferation of spermatogonial stem cells through EGFR in a time-dependent manner [38]. In addition, studies have shown that EGF can regulate the proliferation of Leydig cells and maintain the concentration of testosterone [39]. As shown in Table 2, these targets are upstream or downstream of PI3K-Akt, IL-17, camp, FOXO, MAPK, and other signal pathways. They may play a key role in the treatment of male infertility by PAA.

**Figure 8:**
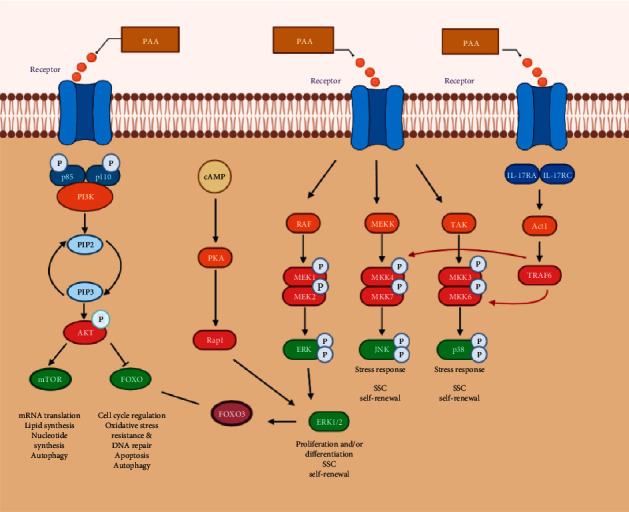
Diagram of signaling pathways associated with infertility.

KEGG enrichment analysis showed that the target pathways of PAA in the treatment of male infertility included the PI3K-Akt, IL-17 (interleukin 17), cAMP, FOXO, MAPK, and other signaling pathways. A mechanism diagram is shown in Figure 8. How these signaling pathways relate to the development or treatment of male infertility is briefly described below. As shown in Figure 6, the PI3K-Akt signaling and cAMP signaling pathways involved the most targets. The PI3K/AKT pathway is an important signaling pathway in the human body. PI3K is a heterodimer that simultaneously exhibits serine/threonine kinase activity and phosphatidylinositol kinase activity. It is composed of the regulatory subunit P85 and the catalytic subunit p110, which is closely related to spermatogenesis and maturation; for example, it affects the proliferation and differentiation of spermatogonial stem cells and the meiosis of spermatocytes [40]. PI3K can be activated not only by tyrosine kinase (receptor tyrosine kinase, RTK) and ras proteins on the cell membrane but also by other proteins. AKT is the direct target gene of PI3K [41, 42]. After PI3K activation, Akt can be activated by phosphorylation of phosphatidylinositol 4-phosphate and phosphatidylinositol 4-diphosphate. Some studies have found that a PI3K-specific inhibitor (LY294002) can significantly improve human sperm motility and the percentage of forward sperm motility [43], and the effect is more obvious in people with asthenospermia [44]. This finding suggests that PI3K negatively regulates the motility of human sperm cells. In addition, PI3K is a negative regulator of autophagy, and autophagy can participate in the regulation of sperm survival and movement. Notably, aflatoxin can induce sperm cells autophagy by inhibiting the PI3K/AKT/mTOR pathway, thereby causing damage to male fertility [45]. FOXOs are located downstream of growth factor and nutrient signaling. In mammals, the FOXOs include Foxo1, Foxo3, and Foxo4, which coordinate various responses, including cell cycle arrest and programmed cell death [46]. FOXO transcription factors are the key nodes at the intersections of many signaling pathways [46]. Previous studies have confirmed that FOXOs are located downstream of the PI3K/AKT signaling pathway and are regulated by Akt-dependent phosphorylation. When AKT is phosphorylated, FOXOs are inhibited [47]. In terms of reproduction, FOXO1 plays an important role in spermatogenesis [48, 49]. FOXO also plays an important role in the self-renewal and differentiation of spermatogonial stem cells (SSCs). When PI3K/AKT is activated, FOXO1 loses its activity, which further inhibits the self-renewal of SSCs [50], indicating that this FOXO plays an important regulatory role in the late stage of spermatogenesis. MAPK belongs to the serine/threonine kinase family. There are three main subfamilies: the ERK, c-Jun N-terminal kinase (JNK), and p38MAPK (MAPK14) subfamilies; this family is thought to be one important determinant of sperm development [51–54]. In rat testes, phosphorylated ERK1/22, JNK1/2, and p38MAPK are localized in SSCs, which play an important role in regulating nutritional supply, maintaining cell connections and supporting mitosis and meiosis of germ cells [57]. The migration of germ cells and the release of sperm require adherens junctions (AJs) and tight junctions (TJs) between Sertoli cells (SC-SC junctions) and Sertoli cells (SC-GC junctions). This requirement makes normal spermatogenesis dependent on SSCs. Activation of the p38MAPK and ERK pathways interferes with the AJs between Sertoli cells, and activation of ERK pathways also affects the dynamics of these two connections, thus affecting the self-renewal of SSCs [55]. Many cells can respond to reproductive toxicants, and many of these responses are mediated by activation of the MAPK pathway; for example, bisphenol A can activate the ERK and JNK signaling pathways to induce apoptosis [56], and under the influence of di-n-butyl-phthalate (an endocrine disruptor), the rat testicular tissue is damaged, semen quality is decreased, and there is an elevated p-ERK1/2 and p-JNK expression in the MAPK pathway, but not p38 MAPK phosphorylation levels [57]. In other experiments, however, p38mpak was suggested to play a major role in Sertoli cell injury [58]. Although existing studies have reported inconsistent conclusions, it has become a fact that MAPKs have a close connection with male infertility, which is in accordance with the findings of the present experiment. Camp is closely related to sperm motility [59]. Specifically, it plays an important role in the initiation, alteration, and maintenance of sperm motility [60]. In recent years, IL-17 has been shown to be a proinflammatory cytokine. Through specific binding with its receptor, it can promote inflammation, the immune response, hematopoiesis, and other processes. A cross-sectional study found that IL-17 levels in the semen of infertile patients were significantly higher than those in the semen of normal men [61].

## 5. Conclusion

In conclusion, this study uses a network pharmacology method to study the therapeutic effects of PAA on male infertility at multiple levels and finds that TP53, MAPK1, IL-6, ANXA1, EGF, EGFR, and other genes may be key targets. The PI3K-Akt, IL-17 cAMP, FOXO, and MAPK signaling pathways may be key pathways that mainly play anti-inflammatory and antioxidant roles and promote cell proliferation and cell ATP production to treat male infertility. A possible molecular mechanism by which this drug pair treats male infertility has been revealed. A limitation of this study is that the pharmacologically active components and targets discovered through network pharmacology are predictions; thus, the results should be verified experimentally. Subsequent research will verify the findings in animal experiments and clinical studies to improve the rationality and scientific basis of clinical PAA application.

## Figures and Tables

**Table 1:** Detailed data of the top 20 targets.

Target	Degree	Betweenness centrality	Closeness centrality	MCODE score
MAPK1	44	0.11909067	0.40682415	3.181818182
TP53	44	0.07271146	0.37349398	3.454545455
AKT1	40	0.10656178	0.4005168	4.169117647
Jun	39	0.04830527	0.38993711	2.759259259
AGT	37	0.07377641	0.3583815	17
RXRA	35	0.10861121	0.40207523	6.222222222
RELA	31	0.0222772	0.3708134	2.675324675
HSP90AA1	31	0.02770788	0.36470588	3.264705882
NCOA1	30	0.03957597	0.37575758	6.222222222
ANXA1	30	0.01792683	0.32460733	17
EDN1	30	0.03558045	0.37530266	11
EGFR	29	0.04140608	0.36172695	8
TNF	29	0.03633951	0.36299766	2.810526316
NR3C1	28	0.0253214	0.38130381	2.685714286
MED1	27	0.03261089	0.36172695	6.222222222
ADCY1	27	0.01606181	0.31155779	17
ESR1	26	0.04038295	0.37621359	2.947368421
IL6	26	0.02207884	0.35107588	6
PPARA	25	0.05492241	0.39340102	6.222222222
VEGFA	25	0.02270293	0.35227273	2.911764706

**Table 2:** Male-infertility-related pathways and involved targets.

Description	Count	Targets
cAMP signaling pathway	18	ACOX1, ADCY1, ADRB2, AKT1, CFTR, CHRM2, CREB1, DRD2, EDN1, EDNRA, FOS, HTR1A, NFKB1, NFKBIA, PPARA, PRKCA, MAPK1, SST
PI3K-Akt signaling pathway	20	AKT1, CCND1, CDK2, CDKN1A, CHRM2, CREB1, EGF, EGFR, HSP90AA1, HSP90AB1, IL4, IL6, KDR, NFKB1, NOS3, PRKCA, MAPK1, RXRA, TP53, FGF23
IL-17 signaling pathway	12	CASP8, FOS, HSP90AA1, HSP90AB1, IFNG, IL1B, IL4, IL6, IL13, NFKB1, NFKBIA, MAPK1
FOXO signaling pathway	11	AKT1, CCND1, CAT, CDK2, CDKN1A, EGF, EGFR, IL6, MAPK1, SLC2A4, TGFB1
MAPK signaling pathway	13	AKT1, EGF, EGFR, FOS, IL1B, IL6, KDR, NFKB1, PRKCA, MAPK1, TGFB1, TP53, FGF23

**Table 3:** Docking results.

Ligand	Receptor (PDB ID)	Lowest binding energy (kJ/mol)
Stigmasterol	EGF (1nql)	−14.0
Stigmasterol	EGFR (5ug9)	−11.5
Quercetin	EGF (1nql)	−11.5
Stigmasterol	ANXA1 (1mcx)	−11.0
Stigmasterol	IL6 (4cni)	−10.0
Stigmasterol	EGFR (5ug9)	−9.3
Quercetin	EGF (1nql)	−9.0
Kaempferol	IL6 (4cni)	−8.8
Kaempferol	TP53 (3d06)	−8.7
Kaempferol	EGF (1nql)	−8.5
Stigmasterol	TP53 (3d06)	−8.3
Stigmasterol	MAPK1 (2waj)	−7.5
Kaempferol	ANXA1 (1mcx)	−7.2
Quercetin	TP53 (3d06)	−6.9
Quercetin	ANXA1 (1mcx)	−5.7
Kaempferol	MAPK1 (2waj)	−5.0
Quercetin	IL6 (4cni)	−3.8
Quercetin	MAPK1 (2waj)	−2.8

## Data Availability

Specific study data are available from the corresponding author upon request.

## Supplementary Materials

Supplementary Materials Table S1: available ingredient and target information of *Astragalus* collected in TCMSP database. Table S2: available ingredient and target information of *Astragalus* collected in BATMAN-TCM database. Table S3: available ingredient and target information of *Angelica* collected in TCMSP database. Table S4: available ingredient and target information of *Angelica* collected in BATMAN-TCM database. Table S5: The drug pair-component-node data of target-disease regulation network.
