# Supplementary material for: Network Pharmacology-Based Strategy to Investigate Pharmacological Mechanisms of the Drug Pair Astragalus-Angelica for Treatment of Male Infertility
Source: Evid Based Complement Alternat Med. 2021 Oct 16;2021:8281506. doi: 10.1155/2021/8281506 (PMC8541871; doi:10.1155/2021/8281506)
Supplement: Supplementary Materials — Table S1: available ingredient and target information of Astragalus collected in TCMSP database. Table S2: available ingredient and target information of Astragalus collected in BATMAN-TCM database. Table S3: available ingredient and target information of Angelica collected in TCMSP database. Table S4: available ingredient and target information of Angelica collected in BATMAN-TCM database. Table S5: The drug pair-component-node data of target-disease regulation network. [file 8281506.f1.zip › 8281506.f1/Table S1. Available ingredient and target information of Astragalus collected in tcmsp database..pdf]

Ingredient	Target
Mairin	PGR
Jaranol	NOS2
Jaranol	PTGS1
Jaranol	AR
Jaranol	SCN5A
Jaranol	PTGS1
Jaranol	ESR2
Jaranol	DPP4
Jaranol	HSP90
Jaranol	CDK2
Jaranol	CHEK1
Jaranol	PRSS1
Jaranol	NCOA2
Jaranol	CALM
(3S,8S,9S,1	PGR
hederagen	PGR
hederagen	NCOA2
hederagen	CHRM3
hederagen	CHRM1
hederagen	GABRA2
hederagen	GABRA3
hederagen	CHRM2
hederagen	ADRA1B
hederagen	GABRA1
hederagen	GRIA2
hederagen	GABRA6
hederagen	GABRA5
hederagen	IGHG1
hederagen	ADH1B
hederagen	ADH1C
hederagen	LYZ
hederagen	N/A
hederagen	PTGS1
hederagen	SCN5A
hederagen	PTGS2
hederagen	RXRA
hederagen	PDE3A
hederagen	SLC6A2
hederagen	?
isorhamne	NOS2
isorhamne	PTGS1
isorhamne	ESR1
isorhamne	AR
isorhamne	PPARG
isorhamne	PTGS2
isorhamne	PTPN1
isorhamne	ESR2
isorhamne	DPP4
isorhamne	MAPK14
isorhamne	GSK3B
isorhamne	HSP90
isorhamne	CDK2
isorhamne	PIK3CG
isorhamne	PRKACA
isorhamne	PRSS1
isorhamne	PIM1
isorhamne	CCNA2

isorhamne	NCOA2
isorhamne	CALM
isorhamne	PYGM
isorhamne	PPARD
isorhamne	CHEK1
isorhamne	AKR
isorhamne	NCOA1
isorhamne	F7
isorhamne	N/A
isorhamne	NOS3
isorhamne	ACHE
isorhamne	GABRA1
isorhamne	MAOB
isorhamne	GRIA2
isorhamne	?
isorhamne	RELA
isorhamne	XDH
isorhamne	NCF1
isorhamne	OLR1
3,9-di-O-r	NOS2
3,9-di-O-r	PTGS1
3,9-di-O-r	CHRM3
3,9-di-O-r	N/A
3,9-di-O-r	CHRM1
3,9-di-O-r	ESR1
3,9-di-O-r	ADRB1
3,9-di-O-r	SCN5A
3,9-di-O-r	PTGS2
3,9-di-O-r	NOS3
3,9-di-O-r	HTR3A
3,9-di-O-r	ADRA2C
3,9-di-O-r	RXRA
3,9-di-O-r	ACHE
3,9-di-O-r	PDE3A
3,9-di-O-r	ADRA1B
3,9-di-O-r	ADRB2
3,9-di-O-r	ADRA1D
3,9-di-O-r	OPRM1
3,9-di-O-r	GABRA1
3,9-di-O-r	PRSS1
3,9-di-O-r	NCOA2
3,9-di-O-r	CALM
5'-hydroxy	N/A
7-O-meth	NOS2
7-O-meth	PTGS1
7-O-meth	DRD1
7-O-meth	CHRM3
7-O-meth	N/A
7-O-meth	KCNH2
7-O-meth	CHRM1
7-O-meth	ESR1
7-O-meth	AR
7-O-meth	ADRB1
7-O-meth	SCN5A
7-O-meth	PPARG
7-O-meth	F10?
7-O-meth	CHRM5
7-O-meth	PTGS2

7-O-meth	NOS3
7-O-meth	ADRA2C
7-O-meth	CHRM4
7-O-meth	RXRA
7-O-meth	OPRD1
7-O-meth	PDE3A
7-O-meth	HTR2A
7-O-meth	ADRA1A
7-O-meth	CHRM2
7-O-meth	ADRA1B
7-O-meth	SLC6A3
7-O-meth	ADRB2
7-O-meth	ADRA1D
7-O-meth	SLC6A4
7-O-meth	ESR2
7-O-meth	GABRA1
7-O-meth	DPP4
7-O-meth	MAPK14
7-O-meth	GSK3B
7-O-meth	HSP90
7-O-meth	CDK2
7-O-meth	CHEK1
7-O-meth	PRKACA
7-O-meth	RXRA
7-O-meth	PRSS1
7-O-meth	PIM1
7-O-meth	CCNA2
7-O-meth	NCOA2
7-O-meth	KCNMA1
7-O-meth	CALM
9,10-dime	PTGS2
9,10-dime	TOP2A
9,10-dime	NCOA2
(6aR,11aR)	NOS2
(6aR,11aR)	PTGS1
(6aR,11aR)	CHRM3
(6aR,11aR)	N/A
(6aR,11aR)	CHRM1
(6aR,11aR)	ESR1
(6aR,11aR)	SCN5A
(6aR,11aR)	PTGS2
(6aR,11aR)	HTR3A
(6aR,11aR)	RXRA
(6aR,11aR)	ACHE
(6aR,11aR)	ADRA1B
(6aR,11aR)	ADRB2
(6aR,11aR)	ADRA1D
(6aR,11aR)	GABRA1
(6aR,11aR)	HSP90
(6aR,11aR)	CHRNA7
(6aR,11aR)	PRSS1
(6aR,11aR)	NCOA2
(6aR,11aR)	NCOA1
(6aR,11aR)	CALM
(6aR,11aR)	CHRM4
Bifendate	PTGS2
Bifendate	KDR
Bifendate	MET

Bifendate	HSP90
Bifendate	KCNMA1
Bifendate	PTGS1
Bifendate	TOP2A
formononetin	NOS2
formononetin	PTGS1
formononetin	CHRM1
formononetin	ESR1
formononetin	AR
formononetin	PPARG
formononetin	PTGS2
formononetin	RXRA
formononetin	PDE3A
formononetin	ADRA1A
formononetin	SLC6A3
formononetin	ADRB2
formononetin	SLC6A4
formononetin	ESR2
formononetin	DPP4
formononetin	MAPK14
formononetin	GSK3B
formononetin	HSP90
formononetin	CDK2
formononetin	MAOB
formononetin	CHEK1
formononetin	PRKACA
formononetin	PRSS1
formononetin	PIM1
formononetin	CCNA2
formononetin	CALM
formononetin	PRKAR1A
formononetin	N/A
formononetin	NOS3
formononetin	ACHE
formononetin	N/A
formononetin	JUN
formononetin	PPARG
formononetin	IL4
formononetin	SIRT1
formononetin	ATP5F1B
formononetin	MT-ND6
formononetin	HSD3B2
formononetin	HSD3B1
isoflavanon	N/A
Calycosin	NOS2
Calycosin	PTGS1
Calycosin	ESR1
Calycosin	AR
Calycosin	PPARG
Calycosin	PTGS2
Calycosin	RXRA
Calycosin	PDE3A
Calycosin	ESR2
Calycosin	DPP4
Calycosin	MAPK14
Calycosin	GSK3B
Calycosin	HSP90
Calycosin	CDK2

Calycosin	CHEK1
Calycosin	PRKACA
Calycosin	PRSS1
Calycosin	PIM1
Calycosin	CCNA2
Calycosin	NCOA2
Calycosin	CALM
Calycosin	ADRB2
kaempfero	NOS2
kaempfero	PTGS1
kaempfero	AR
kaempfero	PPARG
kaempfero	PTGS2
kaempfero	HSP90
kaempfero	PIK3CG
kaempfero	PRKACA
kaempfero	NCOA2
kaempfero	DPP4
kaempfero	PRSS1
kaempfero	PGR
kaempfero	N/A
kaempfero	CHRM1
kaempfero	NOS3
kaempfero	GABRA2
kaempfero	ACHE
kaempfero	SLC6A2
kaempfero	CHRM2
kaempfero	ADRA1B
kaempfero	GABRA1
kaempfero	TOP2A
kaempfero	F7
kaempfero	CALM
kaempfero	RELA
kaempfero	IKBKB
kaempfero	AKT1
kaempfero	BCL2
kaempfero	BAX
kaempfero	TNF
kaempfero	JUN
kaempfero	AHSA1
kaempfero	CASP3
kaempfero	MAPK8
kaempfero	XDH
kaempfero	MMP1
kaempfero	STAT1
kaempfero	CDK1
kaempfero	PPARG
kaempfero	HMOX1
kaempfero	CYP3A4
kaempfero	CYP1A2
kaempfero	CYP1A1
kaempfero	ICAM1
kaempfero	SELE
kaempfero	VCAM1
kaempfero	NR1I2
kaempfero	CYP1B1
kaempfero	ALOX5
kaempfero	HAS2

kaempfero	GSTP1
kaempfero	AHR
kaempfero	PSMD3
kaempfero	SLC2A4
kaempfero	NR1I3
kaempfero	INSR
kaempfero	DIO1
kaempfero	PPP3CA
kaempfero	N/A
kaempfero	GSTM1
kaempfero	GSTM2
kaempfero	AKR1C3
kaempfero	N/A
FA	CDK2
FA	N/A
FA	GSK3B
(3R)-3-(2-	N/A
isomucron	TOP2A
1,7-Dihydr	PTGS2
1,7-Dihydr	RXRA
1,7-Dihydr	HSP90
1,7-Dihydr	PRSS1
quercetin	PTGS1
quercetin	AR
quercetin	PPARG
quercetin	PTGS2
quercetin	HSP90
quercetin	PIK3CG
quercetin	NCOA2
quercetin	DPP4
quercetin	AKR
quercetin	PRSS1
quercetin	TOP2A
quercetin	N/A
quercetin	KCNH2
quercetin	SCN5A
quercetin	F10?
quercetin	ADRB2
quercetin	MMP3
quercetin	PRKACA
quercetin	F7
quercetin	NOS3
quercetin	RXRA
quercetin	ACHE
quercetin	GABRA1
quercetin	MAOB
quercetin	RELA
quercetin	EGFR
quercetin	AKT1
quercetin	VEGFA
quercetin	CCND1
quercetin	BCL2
quercetin	BCL2L1
quercetin	FOS
quercetin	CDKN1A
quercetin	EIF6
quercetin	BAX
quercetin	CASP9

quercetin	PLAU
quercetin	MMP2
quercetin	MMP9
quercetin	MAPK1
quercetin	IL10
quercetin	EGF
quercetin	RB1
quercetin	TNF
quercetin	JUN
quercetin	IL6
quercetin	N/A
