# Supplementary material for: Network Pharmacology-Based Strategy to Investigate Pharmacological Mechanisms of the Drug Pair Astragalus-Angelica for Treatment of Male Infertility
Source: Evid Based Complement Alternat Med. 2021 Oct 16;2021:8281506. doi: 10.1155/2021/8281506 (PMC8541871; doi:10.1155/2021/8281506)
Supplement: Supplementary Materials — Table S1: available ingredient and target information of Astragalus collected in TCMSP database. Table S2: available ingredient and target information of Astragalus collected in BATMAN-TCM database. Table S3: available ingredient and target information of Angelica collected in TCMSP database. Table S4: available ingredient and target information of Angelica collected in BATMAN-TCM database. Table S5: The drug pair-component-node data of target-disease regulation network. [file 8281506.f1.zip › 8281506.f1/Table S2. Available ingredient and target information of Astragalus collected in BATMAN-TCM database.pdf]

Ingredient	Target
Astragalos	CXCR4
Chrysanthe	ADA
Chrysanthe	ITFG2
Chrysanthe	DGUOK
Chrysanthe	XDH
Chrysanthe	ADORA1
Soyasapon	NR3C1
Soyasapon	ATP1A1
Soyasapog	ESR1
Soyasapog	PGR
Soyasapog	AR
Soyasapog	NR3C1
Soyasapog	ANXA1
Astramem	CXCR4
Kaempferc	HSD17B1
Kaempferc	CRYZ
Kaempferc	VKORC1
Kaempferc	NQO1
Astragalos	CXCR4
Astragalos	CXCR4
Sucrose	CXCR4
Sucrose	NFKB2
Sucrose	TNF
Sucrose	MMP9
Sucrose	IFNG
Astragalos	CXCR4
3,5-Dimetl	PDE4A
3,5-Dimetl	PDE3A
3,5-Dimetl	CACNA1C
3,5-Dimetl	TRDMT1
3,5-Dimetl	DHFRL1
3,5-Dimetl	DHFR
3,5-Dimetl	PDE10A
3,5-Dimetl	PDE2A
3,5-Dimetl	TYMS
3,5-Dimetl	SHMT1
3,5-Dimetl	PDE3B
3,5-Dimetl	DMTN
3,5-Dimetl	KCNMA1
3,5-Dimetl	ABCC2
3,5-Dimetl	FOLR2
3,5-Dimetl	PDE5A
3,5-Dimetl	PDE9A
3,5-Dimetl	TBPL1
3,5-Dimetl	DNMT3A
3,5-Dimetl	STUB1
3,5-Dimetl	INSR
3,5-Dimetl	P2RX2
3,5-Dimetl	HTR2A
3,5-Dimetl	SIRT2
3,5-Dimetl	HCN4
3,5-Dimetl	DPYD
3,5-Dimetl	HOXA5
3,5-Dimetl	FBLN1
3,5-Dimetl	SLC22A6
3,5-Dimetl	ASNS
3,5-Dimetl	ADK

3,5-Dimetl	IL1B
3,5-Dimetl	NT5M
3,5-Dimetl	P2RX3
3,5-Dimetl	AURKA
3,5-Dimetl	GAS6
3,5-Dimetl	CMPK2
3,5-Dimetl	PDE1B
3,5-Dimetl	DPYS
3,5-Dimetl	CHRNA3
3,5-Dimetl	HSP90AB1
3,5-Dimetl	CHRNB2
3,5-Dimetl	SLC6A4
3,5-Dimetl	DUT
3,5-Dimetl	RAPGEF2
3,5-Dimetl	ATIC
3,5-Dimetl	AK9
3,5-Dimetl	CDKN1A
3,5-Dimetl	HIF1A
3,5-Dimetl	FOLR1
3,5-Dimetl	RAB8B
3,5-Dimetl	HYAL2
3,5-Dimetl	DNMT3B
3,5-Dimetl	HSP90AA1
3,5-Dimetl	DNMT1
3,5-Dimetl	PARP10
3,5-Dimetl	CHRNB4
3,5-Dimetl	MGMT
3,5-Dimetl	DTYMK
3,5-Dimetl	PDE11A
3,5-Dimetl	HCN2
20-Hexade	PRKCA
20-Hexade	PRKCD
20-Hexade	PTGER4
20-Hexade	PTGER2
20-Hexade	PTGER3
20-Hexade	CD300A
20-Hexade	CHGA
20-Hexade	APOC2
20-Hexade	KIF14
20-Hexade	PRKCB
Adenine	ADORA2A
Adenine	ADORA2B
Adenine	ADORA3
Adenine	ADORA1
Adenine	FBP1
Adenine	PRKAB1
Adenine	ACSS2
Adenine	POLE
Adenine	CBS
Adenine	POLE3
Adenine	ADCY1
Adenine	COMT
Adenine	POLA1
Adenine	DCK
Adenine	RRM2B
Adenine	ADK
Adenine	GNMT
Adenine	PNP

Adenine	PDE4D
Adenine	CBSL
Adenine	RRM2
Adenine	PRKAA1
Adenine	MAT1A
Adenine	CREB1
Adenine	ACSS1
Adenine	PIM1
Adenine	PYGL
Adenine	POLE2
Adenine	HINT1
Adenine	MAT2A
Adenine	AMD1
Adenine	ACSL1
Adenine	PDE4B
Adenine	PRKAB2
Adenine	POLE4
Adenine	RRM1
Adenine	MTAP
Adenine	PRPS1
Astragalos	SLC47A1
Astragalos	CYP3A4
Astragalos	CYP51A1
Astragalos	KCNH2
Astragalos	ATP1A1
Astragalos	MLNR
Astragalos	SLCO1B3
Astragalos	ALB
Astragalos	ABCB1
Astragalos	SLCO1B1
Astragalos	SLC47A1
Astragalos	CYP3A4
Astragalos	CYP51A1
Astragalos	KCNH2
Astragalos	ATP1A1
Astragalos	MLNR
Astragalos	SLCO1B3
Astragalos	ALB
Astragalos	ABCB1
Astragalos	SLCO1B1
Betaine	PPAT
Betaine	NPY2R
Betaine	CTPS1
Betaine	CPS1
Betaine	SST
Betaine	GLUL
Betaine	GOT2
Betaine	SLC1A1
Betaine	ACY3
Betaine	ASPA
Betaine	ADSSL1
Betaine	ASNS
Betaine	DARS2
Betaine	GOT1
Betaine	ASPH
Betaine	SLC25A12
Betaine	DARS
Betaine	ASRGL1

Betaine	RNASE1
Betaine	ASS1
Betaine	ADSS
Betaine	CAD
Betaine	ACY1
Betaine	SLC25A13
Betaine	LYZ
Betaine	PAICS
Betaine	BCAT1
Betaine	VARS
Betaine	TARS
Betaine	THNSL1
Betaine	TARS2
Betaine	PCCB
Betaine	SLC5A6
Betaine	UGCG
Betaine	LIAS
Betaine	TH
Betaine	LIPT1
Betaine	HPD
Betaine	TPH1
Betaine	PAH
Betaine	NOS3
Beta-Sitost	ESR1
Beta-Sitost	PGR
Beta-Sitost	VDR
Beta-Sitost	CYP27B1
Beta-Sitost	GC
Beta-Sitost	SNW1
Beta-Sitost	AR
Beta-Sitost	NR3C1
Beta-Sitost	NFKB1
Beta-Sitost	AKR1C3
Beta-Sitost	CYP24A1
Beta-Sitost	GPBAR1
Beta-Sitost	SNAI2
Beta-Sitost	MED1
Beta-Sitost	SNAI1
Beta-Sitost	CYP3A4
Beta-Sitost	CALB1
Beta-Sitost	FGF23
Beta-Sitost	GFI1
Beta-Sitost	LANCL2
Beta-Sitost	WNT4
Beta-Sitost	TCF3
Beta-Sitost	BAX
Beta-Sitost	KL
Beta-Sitost	CYP2R1
Beta-Sitost	PML
Beta-Sitost	B4GALT1
Beta-Sitost	S100G
Beta-Sitost	CYP27A1
Beta-Sitost	KANK2
Beta-Sitost	IRX5
Beta-Sitost	RXRA
Beta-Sitost	NR1H4
Beta-Sitost	TRIM24
Canavanin	AZIN2

Canavanin	ASL
Canavanin	NOS2
Canavanin	SLC7A3
Canavanin	ASS1
Canavanin	SLC7A1
Canavanin	SLC7A4
Canavanin	NOS3
Canavanin	ARG2
Canavanin	HMOX1
Canavanin	OAZ3
Canavanin	AGMAT
Canavanin	KLF4
Canavanin	GNRH1
Canavanin	ARG1
Canavanin	AZIN1
Canavanin	OTC
Canavanin	SLC1A5
Canavanin	GOT2
Canavanin	GPT2
Canavanin	BAAT
Canavanin	SHMT1
Canavanin	ABAT
Canavanin	SLC1A1
Canavanin	SLC7A2
Canavanin	GCSH
Canavanin	SLC7A8
Canavanin	PPAT
Canavanin	ACY3
Canavanin	ASPA
Canavanin	IARS2
Canavanin	GLRA2
Canavanin	ALAS1
Canavanin	BCAT1
Canavanin	GCAT
Canavanin	SLC38A3
Canavanin	VARS
Canavanin	NFS1
Canavanin	SLC25A15
Canavanin	SLC1A4
Canavanin	ADSSL1
Canavanin	VDAC3
Canavanin	GSS
Canavanin	ASNS
Canavanin	GLRA3
Canavanin	DARS2
Canavanin	GRIN3B
Canavanin	LCMT1
Canavanin	GRIN2A
Canavanin	GOT1
Canavanin	GNMT
Canavanin	GLRB
Canavanin	GLYAT
Canavanin	AARS2
Canavanin	ASPH
Canavanin	GPR18
Canavanin	CTPS1
Canavanin	SLC25A12
Canavanin	PHYKPL

Canavanin	GLYATL1
Canavanin	GATM
Canavanin	LARS
Canavanin	NARS2
Canavanin	TNNC1
Canavanin	DARS
Canavanin	GRIN2C
Canavanin	OAT
Canavanin	GLDC
Canavanin	VDAC2
Canavanin	OAZ2
Canavanin	PIPOX
Canavanin	SLC25A2
Canavanin	GARS
Canavanin	GPT
Canavanin	AARS
Canavanin	SHMT2
Canavanin	ALAS2
Canavanin	VDAC1
Canavanin	SLC6A9
Canavanin	ASRGL1
Canavanin	GLYATL2
Canavanin	SLC32A1
Canavanin	OAZ1
Canavanin	KARS
Canavanin	AGXT
Canavanin	LARS2
Canavanin	RNASE1
Canavanin	ADSS
Canavanin	SLC36A1
Canavanin	ACADSB
Canavanin	SLC6A5
Canavanin	CAD
Canavanin	PCCB
Canavanin	GLUL
Canavanin	AGXT2
Canavanin	IARS
Canavanin	BCAT2
Canavanin	ACY1
Canavanin	KYNU
Canavanin	SLC25A13
Canavanin	GLRA1
Canavanin	LYZ
Canavanin	LCMT2
Canavanin	PAICS
Canavanin	NARS
Canavanin	DGAT2
Canavanin	CYBA
Canavanin	RARS
Canavanin	P2RX2
Canavanin	TPH2
Canavanin	EDN1
Canavanin	SLC24A2
Canavanin	PRLR
Canavanin	HTR2B
Canavanin	PAOX
Canavanin	MTRR
Canavanin	TH

Canavanin	EDN3
Canavanin	IL1B
Canavanin	BAK1
Canavanin	POR
Canavanin	PQLC2
Canavanin	HMBS
Canavanin	TPH1
Canavanin	TAF7
Canavanin	BCL2L11
Canavanin	ECE1
Canavanin	FCER2
Canavanin	NDOR1
Canavanin	IFNG
Canavanin	F2RL1
Canavanin	DDC
Canavanin	PAM
Canavanin	SLC11A1
Canavanin	AIF1
Canavanin	SLC3A1
Canavanin	SLC11A2
Canavanin	MT3
Canavanin	SLC7A7
Canavanin	UROS
Canavanin	CPS1
Canavanin	BAD
Canavanin	LCN2
Canavanin	ODC1
Canavanin	ADSL
Canavanin	NAGS
Canavanin	CERS1
Canavanin	NOS1
Canavanin	EDN2
Choline	PLD1
Choline	PCYT1A
Choline	PCYT1B
Choline	PHOSPHO1
Choline	ACHE
Choline	PLD2
Choline	BCHE
Choline	COLQ
Choline	SLC5A7
Choline	HMOX1
Choline	NRG1
Choline	DMGDH
Choline	SIX3
Choline	RAP1GAP
Choline	CRP
Choline	NAPEPLD
Choline	FSCN1
Choline	PRSS12
Choline	KLF5
Choline	SLC44A4
Choline	ALDH7A1
Choline	DNM3
Choline	PLD4
Choline	ASCL1
Choline	GPLD1
Choline	ENPP6

Choline	CDH8
Choline	PLD3
Choline	PODXL
Choline	FNTA
Choline	AGRN
Choline	CHKA
Choline	CHDH
Choline	ATP8B1
Choline	GRIN1
Gamma-Si	ESR1
Gamma-Si	PGR
Gamma-Si	VDR
Gamma-Si	CYP27B1
Gamma-Si	GC
Gamma-Si	SNW1
Gamma-Si	AR
Gamma-Si	NR3C1
Gamma-Si	NFKB1
Gamma-Si	AKR1C3
Gamma-Si	CYP24A1
Gamma-Si	GPBAR1
Gamma-Si	SNAI2
Gamma-Si	MED1
Gamma-Si	SNAI1
Gamma-Si	CYP3A4
Gamma-Si	CALB1
Gamma-Si	FGF23
Gamma-Si	GFI1
Gamma-Si	LANCL2
Gamma-Si	WNT4
Gamma-Si	TCF3
Gamma-Si	BAX
Gamma-Si	KL
Gamma-Si	CYP2R1
Gamma-Si	PML
Gamma-Si	B4GALT1
Gamma-Si	S100G
Gamma-Si	CYP27A1
Gamma-Si	KANK2
Gamma-Si	IRX5
Gamma-Si	RXRA
Gamma-Si	NR1H4
Gamma-Si	TRIM24
Guanosine	POLA1
Guanosine	ADORA2A
Guanosine	POLB
Guanosine	DNMT1
Guanosine	PNP
Guanosine	ADORA2B
Guanosine	ADORA3
Guanosine	ADORA1
Guanosine	TCN1
Guanosine	MUT
Guanosine	MMAB
Guanosine	MTRR
Guanosine	MTR
Guanosine	CUBN
Guanosine	AMN

Guanosine	MMACHC
Guanosine	MMAA
Guanosine	UHRF1
Guanosine	DNMT3A
Guanosine	POLE
Guanosine	BRCA1
Guanosine	PRKDC
Guanosine	KDM1A
Guanosine	POLG
Guanosine	LIG4
Guanosine	USP7
Guanosine	TCF3
Guanosine	POLD1
Guanosine	PAX5
Guanosine	TRDMT1
Guanosine	TACR2
Guanosine	UHRF2
Guanosine	DNMT3B
Guanosine	CTCF
Guanosine	KDM3A
Guanosine	MGMT
Guanosine	HELLS
Lupeol	VDR
Lupeol	CYP27B1
Lupeol	GC
Lupeol	SNW1
Lupeol	TRPV3
Lupeol	ESR1
Lupeol	PGR
Lupeol	OPRK1
Lupeol	TRPM8
Lupeol	TRPA1
Lupeol	NFKB1
Lupeol	AKR1C3
Lupeol	CYP24A1
Lupeol	GPBAR1
Lupeol	SNAI2
Lupeol	MED1
Lupeol	SNAI1
Lupeol	CYP3A4
Lupeol	CALB1
Lupeol	FGF23
Lupeol	GFI1
Lupeol	LANCL2
Lupeol	WNT4
Lupeol	TCF3
Lupeol	BAX
Lupeol	KL
Lupeol	CYP2R1
Lupeol	PML
Lupeol	B4GALT1
Lupeol	S100G
Lupeol	CYP27A1
Lupeol	KANK2
Lupeol	IRX5
Lupeol	RXRA
Lupeol	NR1H4
Lupeol	TRIM24

Quercetin	HTR2A
Quercetin	HTR2B
Quercetin	ADRA2A
Quercetin	HTR1B
Quercetin	HTR2C
Quercetin	HTR1D
Quercetin	DRD2
Quercetin	DRD4
Quercetin	HTR1A
Quercetin	ADRA2C
Quercetin	CALY
Quercetin	DRD3
Quercetin	DRD5
Quercetin	DRD1
Quercetin	ADRA2B
Uridine	TYMS
Uridine	NT5C2
Uridine	ADK
Uridine	IMPDH1
Uridine	ENPP1
Uridine	POLA1
Uridine	PNP
Uridine	POLB
Uridine	TERT
Uridine	GSR
Uridine	GCDH
Uridine	ERO1B
Uridine	IVD
Uridine	NQO2
Uridine	DPYD
Uridine	POR
Uridine	CYB5R1
Uridine	MAOB
Uridine	FDXR
Uridine	DAO
Uridine	TXNRD1
Uridine	ACADS
Uridine	CYB5R3
Uridine	MAOA
Uridine	IL4I1
Uridine	AIFM1
Uridine	ACOX1
Uridine	ACADM
Uridine	NQO1
Uridine	XDH
Uridine	DNMT1
Uridine	ACAD8
Uridine	NOS1
Uridine	DLD
Uridine	GFER
Uridine	AK5
Uridine	GUK1
Uridine	ANKH
Uridine	ENPP3
Uridine	APRT
Uridine	AK9
Uridine	NUDT12
Uridine	IMPDH2
