# Supplementary material for: Network Pharmacology-Based Strategy to Investigate Pharmacological Mechanisms of the Drug Pair Astragalus-Angelica for Treatment of Male Infertility
Source: Evid Based Complement Alternat Med. 2021 Oct 16;2021:8281506. doi: 10.1155/2021/8281506 (PMC8541871; doi:10.1155/2021/8281506)
Supplement: Supplementary Materials — Table S1: available ingredient and target information of Astragalus collected in TCMSP database. Table S2: available ingredient and target information of Astragalus collected in BATMAN-TCM database. Table S3: available ingredient and target information of Angelica collected in TCMSP database. Table S4: available ingredient and target information of Angelica collected in BATMAN-TCM database. Table S5: The drug pair-component-node data of target-disease regulation network. [file 8281506.f1.zip › 8281506.f1/Table S3. Available ingredient and target information of Angelicacollected in tcmsp database.pdf]

Ingredient	Target
beta-sitosterol	PGR
beta-sitosterol	NCOA2
beta-sitosterol	PTGS1
beta-sitosterol	PTGS2
beta-sitosterol	HSP90
beta-sitosterol	PIK3CG
beta-sitosterol	KCNH2
beta-sitosterol	PRKACA
beta-sitosterol	DRD1
beta-sitosterol	CHRM3
beta-sitosterol	CHRM1
beta-sitosterol	SCN5A
beta-sitosterol	GABRA2
beta-sitosterol	CHRM4
beta-sitosterol	PDE3A
beta-sitosterol	HTR2A
beta-sitosterol	GABRA5
beta-sitosterol	ADRA1A
beta-sitosterol	GABRA3
beta-sitosterol	CHRM2
beta-sitosterol	ADRA1B
beta-sitosterol	ADRB2
beta-sitosterol	CHRNA2
beta-sitosterol	SLC6A4
beta-sitosterol	OPRM1
beta-sitosterol	GABRA1
beta-sitosterol	CHRNA7
beta-sitosterol	N/A
beta-sitosterol	BCL2
beta-sitosterol	BAX
beta-sitosterol	CASP9
beta-sitosterol	JUN
beta-sitosterol	CASP3
beta-sitosterol	CASP8
beta-sitosterol	PRKCA
beta-sitosterol	TGFB1
beta-sitosterol	PON1
beta-sitosterol	MAP2
Stigmasterol	PGR
Stigmasterol	NR3C2
Stigmasterol	NCOA2
Stigmasterol	ADH1C
Stigmasterol	IGHG1
Stigmasterol	RXRA
Stigmasterol	NCOA1
Stigmasterol	PTGS1
Stigmasterol	PTGS2
Stigmasterol	ADRA2A
Stigmasterol	SLC6A2
Stigmasterol	SLC6A3
Stigmasterol	ADRB2
Stigmasterol	AKR
Stigmasterol	PLAU
Stigmasterol	LTA4H
Stigmasterol	MAOB
Stigmasterol	MAOA
Stigmasterol	PRKACA

Stigmasterol	CTRB1
Stigmasterol	CHRM3
Stigmasterol	CHRM1
Stigmasterol	ADRB1
Stigmasterol	SCN5A
Stigmasterol	HTR2A
Stigmasterol	ADRA1A
Stigmasterol	GABRA3
Stigmasterol	CHRM2
Stigmasterol	ADRA1B
Stigmasterol	GABRA1
Stigmasterol	CHRNA7
senkyunolide-C	PTGS1
senkyunolide-C	CHRM1
senkyunolide-C	PTGS2
senkyunolide-C	CHRM2
senkyunolide-C	ADRA2B
senkyunolide-C	ADRB2
senkyunolide-C	SLC6A4
senkyunolide-C	GABRA1
senkyunolide-C	MAOB
senkyunolide-C	PRKACA
senkyunolide-C	PKIA
senkyunolide-D	PTGS2
senkyunolide-D	NOS3
senkyunolide-D	GABRA1
senkyunolide-D	GRIA2
senkyunolide-D	GABRA6
senkyunolide-E	PTGS1
senkyunolide-E	CHRM1
senkyunolide-E	SCN5A
senkyunolide-E	PTGS2
senkyunolide-E	SLC6A2
senkyunolide-E	ADRA2B
senkyunolide-E	ADRB2
senkyunolide-E	SLC6A4
senkyunolide-E	GABRA1
senkyunolide-E	MAOB
senkyunolide-E	PRKACA
FERULIC ACID (CIS)	PTGS1
FERULIC ACID (CIS)	PTGS2
FERULIC ACID (CIS)	NOS3
FERULIC ACID (CIS)	ADRA2A
FERULIC ACID (CIS)	SLC6A2
FERULIC ACID (CIS)	ADRA2B
FERULIC ACID (CIS)	SLC6A3
FERULIC ACID (CIS)	ADRB2
FERULIC ACID (CIS)	LTA4H
FERULIC ACID (CIS)	MAOB
FERULIC ACID (CIS)	MAOA
FERULIC ACID (CIS)	PRKACA
FERULIC ACID (CIS)	CTRB1
FERULIC ACID (CIS)	CHRM2
beta-Chamigrene	PTGS2
beta-Chamigrene	SLC6A2
beta-Chamigrene	MAOB
beta-Chamigrene	NCOA2
beta-Chamigrene	PTGS1

beta-Chamigrene	CHRM3
beta-Chamigrene	CHRM1
beta-Chamigrene	GABRA2
beta-Chamigrene	RXRA
beta-Chamigrene	GABRA3
beta-Chamigrene	CHRM2
beta-Chamigrene	ADRA1B
beta-Chamigrene	GABRA1
beta-Chamigrene	GABRA6
beta-Chamigrene	CHRNA2
beta-Chamigrene	CHRNA7
beta-Chamigrene	GABRA5
beta-Chamigrene	ADRA1A
beta-Chamigrene	GABRA4
()-Cuparene	CHRM3
()-Cuparene	N/A
()-Cuparene	CHRM1
()-Cuparene	GABRA2
()-Cuparene	ACHE
()-Cuparene	GABRA5
()-Cuparene	GABRA3
()-Cuparene	CHRM2
()-Cuparene	ADRA1B
()-Cuparene	GABRA1
()-Cuparene	DPP4
()-Cuparene	GRIA2
()-Cuparene	GABRA6
()-Cuparene	PTGS1
()-Cuparene	DRD1
()-Cuparene	SCN5A
()-Cuparene	PTGS2
()-Cuparene	RXRA
()-Cuparene	SLC6A2
()-Cuparene	ADRA1A
()-Cuparene	SLC6A3
()-Cuparene	ADRB2
()-Cuparene	SLC6A4
()-Cuparene	PDE3A
()-Cuparene	ADRA1D
()-Cuparene	CHRNA2
()-Cuparene	ESR1
()-Cuparene	ADRA2A
vanillin	MAOB
vanillin	MAOA
vanillin	LYZ
vanillin	N/A
vanillin	PTGS2
vanillin	GABRA1
vanillin	ADH1C
vanillin	N/A
vanillin	MMP9
vanillin	MAPK1
vanillin	JUN
