# Supplementary material for: Network Pharmacology-Based Strategy to Investigate Pharmacological Mechanisms of the Drug Pair Astragalus-Angelica for Treatment of Male Infertility
Source: Evid Based Complement Alternat Med. 2021 Oct 16;2021:8281506. doi: 10.1155/2021/8281506 (PMC8541871; doi:10.1155/2021/8281506)
Supplement: Supplementary Materials — Table S1: available ingredient and target information of Astragalus collected in TCMSP database. Table S2: available ingredient and target information of Astragalus collected in BATMAN-TCM database. Table S3: available ingredient and target information of Angelica collected in TCMSP database. Table S4: available ingredient and target information of Angelica collected in BATMAN-TCM database. Table S5: The drug pair-component-node data of target-disease regulation network. [file 8281506.f1.zip › 8281506.f1/Table S4. Available ingredient and target information of Angelicacollected in BATMAN-TCM database.pdf]

Ingredient	Target
Dimethyl P	SCN10A
Dimethyl P	ACHE
Dimethyl P	BCHE
Dimethyl P	COLQ
Dimethyl P	SCN1A
Dimethyl P	GABRB1
Dimethyl P	GLRA2
Dimethyl P	GLRA3
Dimethyl P	GLRB
Dimethyl P	DBH
Dimethyl P	ALDH2
Dimethyl P	GLRA1
Beta-Myrc	KCND1
Beta-Myrc	KCNA3
Beta-Myrc	PRKAB1
Beta-Myrc	ADH1A
Beta-Myrc	KCNA10
Beta-Myrc	GAMT
Beta-Myrc	KCNC3
Beta-Myrc	KCNA1
Beta-Myrc	KCNA2
Beta-Myrc	TPO
Beta-Myrc	CAT
Beta-Myrc	KCNB1
Beta-Myrc	ADH1B
Beta-Myrc	DLG4
Beta-Myrc	KCNC2
Beta-Myrc	KCNC1
Beta-Myrc	ADH1C
Beta-Myrc	KCNB2
Beta-Myrc	KCNA5
Beta-Myrc	RNASE1
Beta-Myrc	KCND2
Beta-Myrc	ALDH2
Beta-Myrc	KCNA4
Beta-Myrc	KCNA7
Beta-Myrc	KCNA6
Beta-Myrc	KCND3
Beta-Myrc	GUCY1B3
Beta-Myrc	GATM
Beta-Myrc	KCNK4
Beta-Myrc	IYD
Beta-Myrc	KCNQ1
Ethanol	CACNB1
Ethanol	GRIA4
Ethanol	GABRA2
Ethanol	CACNG2
Ethanol	GABRD
Ethanol	CHRNA4
Ethanol	CHRNB3
Ethanol	GABRB1
Ethanol	GRIA1
Ethanol	GLRA2
Ethanol	KCNJ3
Ethanol	GABRG3
Ethanol	VCAM1
Ethanol	CHRNA7

Ethanol	KCNJ6
Ethanol	GABRE
Ethanol	GABRA3
Ethanol	SLC29A1
Ethanol	CACNA1S
Ethanol	GABRG1
Ethanol	GABRP
Ethanol	CHRNA10
Ethanol	GRIA2
Ethanol	GABRA4
Ethanol	CHRNA3
Ethanol	GABRB2
Ethanol	CACNA1C
Ethanol	CHRNB2
Ethanol	CACNA1D
Ethanol	HTR3D
Ethanol	GABRQ
Ethanol	CHRNA2
Ethanol	CHRNA6
Ethanol	GABRB3
Ethanol	KCNJ5
Ethanol	GRIN3A
Ethanol	HTR3B
Ethanol	GRIA3
Ethanol	GABRA5
Ethanol	SLC29A2
Ethanol	HTR3E
Ethanol	GABRA6
Ethanol	HTR3A
Ethanol	GABRA1
Ethanol	CHRNA9
Ethanol	CHRFAM7A
Ethanol	CHRNA5
Ethanol	CHRNB4
Ethanol	KCNJ9
Ethanol	HTR3C
Ethanol	GLRA1
Ethanol	CACNG1
Ethanol	ADAM8
Ethanol	SLC28A3
Ethanol	ADRA1B
Ethanol	FOXL2
Ethanol	DMTN
Ethanol	KCNMA1
Tetradecar	KCND1
Tetradecar	GAMT
Tetradecar	CAT
Tetradecar	KCNC1
Tetradecar	KCND2
Tetradecar	KCND3
Tetradecar	KCNQ1
Tetradecar	KCNA3
Tetradecar	KCNC3
Tetradecar	KCNB1
Tetradecar	ADH1C
Tetradecar	ALDH2
Tetradecar	GUCY1B3
Tetradecar	PRKAB1

Tetradecar	KCNA1
Tetradecar	ADH1B
Tetradecar	KCNB2
Tetradecar	KCNA4
Tetradecar	GATM
Tetradecar	ADH1A
Tetradecar	KCNA2
Tetradecar	DLG4
Tetradecar	KCNA5
Tetradecar	KCNA7
Tetradecar	KCNK4
Tetradecar	KCNA10
Tetradecar	TPO
Tetradecar	KCNC2
Tetradecar	RNASE1
Tetradecar	KCNA6
Tetradecar	IYD
Carvacrol	GABRA2
Carvacrol	TNF
Carvacrol	GABRP
Carvacrol	GABRQ
Carvacrol	PDE4B
Carvacrol	COLQ
Carvacrol	SLC6A4
Carvacrol	DRD3
Carvacrol	GABRD
Carvacrol	GABRE
Carvacrol	GABRA4
Carvacrol	GABRB3
Carvacrol	BCHE
Carvacrol	LTA
Carvacrol	DRD4
Carvacrol	ALOX5
Carvacrol	PDE3A
Carvacrol	SCN2A
Carvacrol	GABRB2
Carvacrol	GABRA5
Carvacrol	GABRG2
Carvacrol	RXRA
Carvacrol	HTR7
Carvacrol	DRD5
Carvacrol	GABRB1
Carvacrol	GABRA3
Carvacrol	ACHE
Carvacrol	GABRA6
Carvacrol	PDE3B
Carvacrol	SLC6A3
Carvacrol	HTR1A
Carvacrol	DRD1
Carvacrol	GABRG3
Carvacrol	GABRG1
Carvacrol	SCN4A
Carvacrol	GABRA1
Carvacrol	PDE4D
Carvacrol	DRD2
Carvacrol	DBH
Carvacrol	SLC6A2
2',4'-Dihyd	PTGS1

2',4'-Dihyd	PTGS2
Uridine	TYMS
Uridine	POLA1
Uridine	NT5C2
Uridine	PNP
Uridine	ADK
Uridine	POLB
Uridine	IMPDH1
Uridine	TERT
Uridine	ENPP1
Beta-Elem	KCND1
Beta-Elem	GAMT
Beta-Elem	CAT
Beta-Elem	KCNC1
Beta-Elem	KCND2
Beta-Elem	KCND3
Beta-Elem	KCNQ1
Beta-Elem	KCNA3
Beta-Elem	KCNC3
Beta-Elem	KCNB1
Beta-Elem	ADH1C
Beta-Elem	ALDH2
Beta-Elem	GUCY1B3
Beta-Elem	PRKAB1
Beta-Elem	KCNA1
Beta-Elem	ADH1B
Beta-Elem	KCNB2
Beta-Elem	KCNA4
Beta-Elem	GATM
Beta-Elem	ADH1A
Beta-Elem	KCNA2
Beta-Elem	DLG4
Beta-Elem	KCNA5
Beta-Elem	KCNA7
Beta-Elem	KCNK4
Beta-Elem	KCNA10
Beta-Elem	TPO
Beta-Elem	KCNC2
Beta-Elem	RNASE1
Beta-Elem	KCNA6
Beta-Elem	IYD
Azelaic Aci	AKR1D1
Azelaic Aci	ABAT
Azelaic Aci	COX5B
Azelaic Aci	SCN7A
Azelaic Aci	COX5A
Azelaic Aci	COX4I1
Azelaic Aci	SCN4A
Azelaic Aci	CES1
Azelaic Aci	COX2
Azelaic Aci	SUCLG2
Azelaic Aci	SLC25A10
Azelaic Aci	SUCNR1
Azelaic Aci	OXCT1
Azelaic Aci	SDHA
Azelaic Aci	DCT
Azelaic Aci	JMJD6
Azelaic Aci	HSD17B11

Azelaic AciP4HB
Azelaic AciGRIN3B
Azelaic AciCACNA1B
Azelaic AciGRIN3A
Azelaic AciGRIN1
Azelaic AciTYR
Azelaic AciSCN1A
Azelaic AciCOX7C
Azelaic AciSCN5A
Azelaic AciCOX3
Azelaic AciHDAC9
Azelaic AciSCN4B
Azelaic AciACADSB
Azelaic AciOGDH
Azelaic AciSLC13A2
Azelaic AciP3H3
Azelaic AciSLC13A3
Azelaic AciPLOD3
Azelaic AciP4HA2
Azelaic AciAKR1C3
Azelaic AciSLC13A4
Azelaic AciTYRP1
Azelaic AciCACNA2D1
Azelaic AciCACNA2D2
Azelaic AciGRIN2C
Azelaic AciSLC7A1
Azelaic AciSRD5A2
Azelaic AciESRRG
Azelaic AciCOX1
Azelaic AciSCN10A
Azelaic AciAR
Azelaic AciPLA2G1B
Azelaic AciADH1C
Azelaic AciSCN8A
Azelaic AciHDAC2
Azelaic AciSLC13A1
Azelaic AciOXCT2
Azelaic AciASPH
Azelaic AciP4HA1
Azelaic AciSUCLA2
Azelaic AciCRTAP
Azelaic AciSDHAF2
Azelaic AciHIF1AN
Azelaic AciPLAT
Azelaic AciGRIN2A
Azelaic AciGRIN2B
Azelaic AciSLC7A4
Azelaic AciSCN11A
Azelaic AciSCN3B
Azelaic AciSCN3A
Azelaic AciALDH5A1
Azelaic AciCOX7A1
Azelaic AciCOX6A2
Azelaic AciCOX6B1
Azelaic AciCOX7B
Azelaic AciCOX8A
Azelaic AciSUCLG1
Azelaic AciTMLHE

Azelaic Aci	BBOX1
Azelaic Aci	SDHC
Azelaic Aci	SDHD
Azelaic Aci	SLIT2
Azelaic Aci	SLC1A3
Azelaic Aci	UROS
Azelaic Aci	SLC7A2
Azelaic Aci	TOP1
Azelaic Aci	SLC7A3
Azelaic Aci	GRIN2D
Azelaic Aci	SCN2B
Azelaic Aci	COX6C
Azelaic Aci	AKR1C2
Azelaic Aci	SCN2A
Azelaic Aci	FECH
Azelaic Aci	SCN9A
Azelaic Aci	FABP6
Azelaic Aci	SCN1B
Azelaic Aci	NR1H4
Azelaic Aci	HSD17B6
Azelaic Aci	PLOD1
Azelaic Aci	SDHB
Azelaic Aci	P3H2
Azelaic Aci	P3H1
Azelaic Aci	ACO2
Azelaic Aci	SALL1
Azelaic Aci	SLC13A5
Azelaic Aci	CACNA1A
Azelaic Aci	PLG
Azelaic Aci	KARS
Azelaic Aci	ADORA1
2-Methyl-Dodecane-5-One	
2-Methyl-	SCN11A
2-Methyl-	SCN3A
2-Methyl-	ALDH5A1
2-Methyl-	SCN4A
2-Methyl-	SCN1B
2-Methyl-	SCN2B
2-Methyl-	SCN7A
2-Methyl-	SCN2A
2-Methyl-	SCN4B
2-Methyl-	OGDH
2-Methyl-	ABAT
2-Methyl-	SCN5A
2-Methyl-	HDAC9
2-Methyl-	SRD5A2
2-Methyl-	HDAC2
2-Methyl-	SCN1A
2-Methyl-	SCN10A
2-Methyl-	TYR
2-Methyl-	ACADSB
2-Methyl-	SCN3B
2-Methyl-	AKR1D1
2-Methyl-	SCN9A
2-Methyl-	SCN8A
24,24-Dim	ESR1
24,24-Dim	SNW1
24,24-Dim	PGR

24,24-Dim	SRD5A1
24,24-Dim	VDR
24,24-Dim	AR
24,24-Dim	CYP27B1
24,24-Dim	NR3C1
24,24-Dim	GC
24,24-Dim	ANXA1
Dimethyl A	NPR1
Dimethyl A	ESRRG
Dimethyl A	COX1
Dimethyl A	SCN10A
Dimethyl A	COX3
Dimethyl A	HDAC9
Dimethyl A	SCN4A
Dimethyl A	CES1
Dimethyl A	SCN1B
Dimethyl A	NR1H4
Dimethyl A	SCN11A
Dimethyl A	SCN3B
Dimethyl A	SCN3A
Dimethyl A	AKR1D1
Dimethyl A	AR
Dimethyl A	PLA2G1B
Dimethyl A	SCN4B
Dimethyl A	SRD5A2
Dimethyl A	COX2
Dimethyl A	SCN2B
Dimethyl A	COX6C
Dimethyl A	AKR1C2
Dimethyl A	ALDH5A1
Dimethyl A	COX7A1
Dimethyl A	COX6A2
Dimethyl A	ADH1C
Dimethyl A	ACADSB
Dimethyl A	OGDH
Dimethyl A	ABAT
Dimethyl A	COX5B
Dimethyl A	SCN7A
Dimethyl A	SCN2A
Dimethyl A	FECH
Dimethyl A	TYR
Dimethyl A	COX6B1
Dimethyl A	SCN8A
Dimethyl A	HDAC2
Dimethyl A	SCN1A
Dimethyl A	COX7C
Dimethyl A	SCN5A
Dimethyl A	COX5A
Dimethyl A	COX4I1
Dimethyl A	SCN9A
Dimethyl A	FABP6
Dimethyl A	COX7B
Dimethyl A	COX8A
Guanosine	POLA1
Guanosine	ADORA2B
Guanosine	ADORA2A
Guanosine	ADORA3
Guanosine	POLB

Guanosine	ADORA1
Guanosine	DNMT1
Guanosine	PNP
Dodecenoi	RXRB
Dodecenoi	NR0B1
Dodecenoi	CKM
Dodecenoi	PPARD
Dodecenoi	PTGS2
Dodecenoi	CKB
Dodecenoi	APOA2
Dodecenoi	IL13
Dodecenoi	COL27A1
Dodecenoi	SLC8A1
Dodecenoi	TRPV1
Dodecenoi	RARB
Dodecenoi	SLC6A8
Dodecenoi	CKMT1A
Dodecenoi	GPRC5A
Dodecenoi	ALDH3A1
Dodecenoi	GATM
Dodecenoi	SERPINB7
Dodecenoi	GAMT
Dodecenoi	RARG
Dodecenoi	RXRG
Dodecenoi	PTGS1
Dodecenoi	ELOVL4
Dodecenoi	RXRA
Dodecenoi	IL1B
Dodecenoi	SC5D
Dodecenoi	CKMT1B
Dodecenoi	PPARG
Dodecenoi	FADS2
Dodecenoi	ACSL4
Dodecenoi	ALDH1A1
Dodecenoi	ALDH1A2
Dodecenoi	TNF
Dodecenoi	SLC16A12
Dodecenoi	RARRES1
Dodecenoi	FFAR1
Dodecenoi	FADS1
Dodecenoi	CKMT2
Dodecenoi	RARA
Dodecenoi	ACSL3
Dodecenoi	SLC9A1
Dodecenoi	LPCAT1
Dimethyl-E	SCN11A
Dimethyl-E	SCN1A
Dimethyl-E	SCN3A
Dimethyl-E	SCN7A
Dimethyl-E	SLC13A3
Dimethyl-E	BBOX1
Dimethyl-E	OXCT1
Dimethyl-E	SDHC
Dimethyl-E	P3H2
Dimethyl-E	HDAC2
Dimethyl-E	CRTAP
Dimethyl-E	SDHAF2
Dimethyl-E	HIF1AN

Dimethyl-E	SCN2B
Dimethyl-E	SCN3B
Dimethyl-E	SLC25A10
Dimethyl-E	PLOD1
Dimethyl-E	AKR1D1
Dimethyl-E	SDHB
Dimethyl-E	SCN4A
Dimethyl-E	SRD5A2
Dimethyl-E	SDHA
Dimethyl-E	SDHD
Dimethyl-E	SLIT2
Dimethyl-E	SLC1A3
Dimethyl-E	UROS
Dimethyl-E	SUCLG2
Dimethyl-E	SLC13A1
Dimethyl-E	P3H3
Dimethyl-E	SCN5A
Dimethyl-E	ALDH5A1
Dimethyl-E	HDAC9
Dimethyl-E	SCN4B
Dimethyl-E	ACADSB
Dimethyl-E	OGDH
Dimethyl-E	P3H1
Dimethyl-E	ACO2
Dimethyl-E	SALL1
Dimethyl-E	SLC13A5
Dimethyl-E	SLC13A2
Dimethyl-E	SUCLG1
Dimethyl-E	OXCT2
Dimethyl-E	SCN10A
Dimethyl-E	SCN2A
Dimethyl-E	TYR
Dimethyl-E	PLOD3
Dimethyl-E	SCN8A
Dimethyl-E	P4HA2
Dimethyl-E	DCT
Dimethyl-E	JMJD6
Dimethyl-E	HSD17B11
Dimethyl-E	P4HB
Dimethyl-E	ABAT
Dimethyl-E	HSD17B6
Dimethyl-E	TMLHE
Dimethyl-E	SUCNR1
Dimethyl-E	ASPH
Dimethyl-E	SCN9A
Dimethyl-E	P4HA1
Dimethyl-E	SCN1B
Dimethyl-E	SUCLA2
Dimethyl-E	AKR1C3
Dimethyl-E	SLC13A4
Dimethyl-E	TYRP1
4-Ethylreso	GABRA2
4-Ethylreso	TNF
4-Ethylreso	GABRP
4-Ethylreso	GABRQ
4-Ethylreso	PDE4B
4-Ethylreso	COLQ
4-Ethylreso	SLC6A4

4-Ethylres	DRD3
4-Ethylres	GABRD
4-Ethylres	GABRE
4-Ethylres	GABRA4
4-Ethylres	GABRB3
4-Ethylres	BCHE
4-Ethylres	LTA
4-Ethylres	DRD4
4-Ethylres	DRD5
4-Ethylres	PDE3A
4-Ethylres	SCN2A
4-Ethylres	GABRB2
4-Ethylres	GABRA5
4-Ethylres	GABRG2
4-Ethylres	RXRA
4-Ethylres	HTR7
4-Ethylres	DRD1
4-Ethylres	GABRB1
4-Ethylres	GABRA3
4-Ethylres	ACHE
4-Ethylres	GABRA6
4-Ethylres	PDE3B
4-Ethylres	SLC6A3
4-Ethylres	HTR1A
4-Ethylres	SLC6A2
4-Ethylres	GABRG3
4-Ethylres	GABRG1
4-Ethylres	SCN4A
4-Ethylres	GABRA1
4-Ethylres	PDE4D
4-Ethylres	DRD2
4-Ethylres	DBH
Dimethyl S	NPR1
Dimethyl S	ESRRG
Dimethyl S	COX1
Dimethyl S	SCN10A
Dimethyl S	COX3
Dimethyl S	HDAC9
Dimethyl S	SCN4A
Dimethyl S	CES1
Dimethyl S	SCN1B
Dimethyl S	NR1H4
Dimethyl S	SCN11A
Dimethyl S	SCN3B
Dimethyl S	SCN3A
Dimethyl S	AKR1D1
Dimethyl S	AR
Dimethyl S	PLA2G1B
Dimethyl S	SCN4B
Dimethyl S	SRD5A2
Dimethyl S	COX2
Dimethyl S	SCN2B
Dimethyl S	COX6C
Dimethyl S	AKR1C2
Dimethyl S	ALDH5A1
Dimethyl S	COX7A1
Dimethyl S	COX6A2
Dimethyl S	ADH1C

Dimethyl S	ACADSB
Dimethyl S	OGDH
Dimethyl S	ABAT
Dimethyl S	COX5B
Dimethyl S	SCN7A
Dimethyl S	SCN2A
Dimethyl S	FECH
Dimethyl S	TYR
Dimethyl S	COX6B1
Dimethyl S	SCN8A
Dimethyl S	HDAC2
Dimethyl S	SCN1A
Dimethyl S	COX7C
Dimethyl S	SCN5A
Dimethyl S	COX5A
Dimethyl S	COX4I1
Dimethyl S	SCN9A
Dimethyl S	FABP6
Dimethyl S	COX7B
Dimethyl S	COX8A
Phenylacet	PLAT
Phenylacet	CFTR
Phenylacet	HCAR3
Phenylacet	IL13
Phenylacet	CXCR1
Phenylacet	THBD
Phenylacet	PTGS1
Phenylacet	NNMT
Phenylacet	C1QTNF3
Phenylacet	PPARG
Phenylacet	PTGS2
Phenylacet	APOA2
Phenylacet	KCNE2
Phenylacet	BCL2
Phenylacet	HCAR2
Phenylacet	TCAF1
Phenylacet	ANXA1
Phenylacet	FABP2
Phenylacet	QPRT
Phenylacet	IL1B
Phenylacet	SERPINB7
1-Tridecer	KCND1
1-Tridecer	GAMT
1-Tridecer	CAT
1-Tridecer	KCNC1
1-Tridecer	KCND2
1-Tridecer	KCND3
1-Tridecer	KCNQ1
1-Tridecer	KCNA3
1-Tridecer	KCNC3
1-Tridecer	KCNB1
1-Tridecer	ADH1C
1-Tridecer	ALDH2
1-Tridecer	GUCY1B3
1-Tridecer	PRKAB1
1-Tridecer	KCNA1
1-Tridecer	ADH1B
1-Tridecer	KCNB2

1-Tridecer	KCNA4
1-Tridecer	GATM
1-Tridecer	ADH1A
1-Tridecer	KCNA2
1-Tridecer	DLG4
1-Tridecer	KCNA5
1-Tridecer	KCNA7
1-Tridecer	KCNK4
1-Tridecer	KCNA10
1-Tridecer	TPO
1-Tridecer	KCNC2
1-Tridecer	RNASE1
1-Tridecer	KCNA6
1-Tridecer	IYD
Sebiferic A	RXRB
Sebiferic A	NR0B1
Sebiferic A	CKM
Sebiferic A	PPARD
Sebiferic A	PTGS2
Sebiferic A	CKB
Sebiferic A	APOA2
Sebiferic A	IL13
Sebiferic A	COL27A1
Sebiferic A	SCN1A
Sebiferic A	COX7C
Sebiferic A	SCN7A
Sebiferic A	SCN2A
Sebiferic A	FECH
Sebiferic A	COX6A2
Sebiferic A	ADH1C
Sebiferic A	ACADSB
Sebiferic A	OGDH
Sebiferic A	SLC8A1
Sebiferic A	TRPV1
Sebiferic A	RARB
Sebiferic A	SLC6A8
Sebiferic A	CKMT1A
Sebiferic A	GPRC5A
Sebiferic A	ALDH3A1
Sebiferic A	GATM
Sebiferic A	SERPINB7
Sebiferic A	ESRRG
Sebiferic A	COX1
Sebiferic A	SCN5A
Sebiferic A	COX5A
Sebiferic A	PLG
Sebiferic A	TYR
Sebiferic A	COX6B1
Sebiferic A	SCN8A
Sebiferic A	HDAC2
Sebiferic A	GAMT
Sebiferic A	RARG
Sebiferic A	RXRG
Sebiferic A	PTGS1
Sebiferic A	ELOVL4
Sebiferic A	RXRA
Sebiferic A	IL1B
Sebiferic A	SC5D

Sebiferic A	SCN11A
Sebiferic A	SCN3B
Sebiferic A	F12
Sebiferic A	SCN10A
Sebiferic A	COX3
Sebiferic A	COX4I1
Sebiferic A	SCN9A
Sebiferic A	FABP6
Sebiferic A	COX7B
Sebiferic A	COX8A
Sebiferic A	CKMT1B
Sebiferic A	PPARG
Sebiferic A	FADS2
Sebiferic A	ACSL4
Sebiferic A	ALDH1A1
Sebiferic A	ALDH1A2
Sebiferic A	TNF
Sebiferic A	SLC16A12
Sebiferic A	SCN2B
Sebiferic A	COX6C
Sebiferic A	SCN3A
Sebiferic A	AKR1D1
Sebiferic A	AR
Sebiferic A	HDAC9
Sebiferic A	SCN4A
Sebiferic A	CES1
Sebiferic A	SCN1B
Sebiferic A	NR1H4
Sebiferic A	RARRES1
Sebiferic A	FFAR1
Sebiferic A	FADS1
Sebiferic A	CKMT2
Sebiferic A	RARA
Sebiferic A	ACSL3
Sebiferic A	SLC9A1
Sebiferic A	LPCAT1
Sebiferic A	ABAT
Sebiferic A	COX5B
Sebiferic A	AKR1C2
Sebiferic A	ALDH5A1
Sebiferic A	COX7A1
Sebiferic A	PLA2G1B
Sebiferic A	SCN4B
Sebiferic A	SRD5A2
Sebiferic A	COX2
Alpha-Ter	ESR1
Alpha-Ter	ANXA1
Alpha-Ter	PGR
Alpha-Ter	VDR
Alpha-Ter	WNT4
Alpha-Ter	AR
Alpha-Ter	NR3C1
Scopoletin	COMT
Angelicin	SOAT1
Angelicin	ABCC2
Angelicin	ACSS2
Angelicin	TNK2
Angelicin	ABCC9

Angelicin	ASS1
Angelicin	APAF1
Angelicin	ABCG1
Angelicin	GC
Angelicin	ANPEP
Angelicin	AKT1
Angelicin	ACVRL1
Angelicin	ASNS
Angelicin	ACSS1
Angelicin	ACVR1B
Angelicin	ABCC8
Angelicin	ARAF
Angelicin	SNW1
Angelicin	NPC1L1
Angelicin	ACVR1
Angelicin	ADCY1
Angelicin	ABL2
Angelicin	ABL1
Angelicin	ACSL1
Angelicin	ALK
Angelicin	SLC25A4
Angelicin	AR
Angelicin	ESR1
Angelicin	ASNA1
Angelicin	NT5C2
Angelicin	ADRBK1
Angelicin	AFG3L2
Angelicin	ABCA1
Angelicin	ABCB11
Angelicin	VDR
Angelicin	NR3C1
Angelicin	PGR
Angelicin	ADRBK2
Angelicin	AMHR2
Angelicin	PRKAA1
Angelicin	CDK15
Angelicin	NAE1
Angelicin	ABCB1
Angelicin	CYP27B1
Ethyl-P-M	RXRB
Ethyl-P-M	RARA
Ethyl-P-M	RARG
Ethyl-P-M	RXRA
Ethyl-P-M	RARB
Ethyl-P-M	TNF
Ethyl-P-M	RXRG
Ethyl-P-M	COL27A1
Ethyl-P-M	RBP1
Decanoic A	AKR1D1
Decanoic A	ABAT
Decanoic A	COX5B
Decanoic A	SCN7A
Decanoic A	COX5A
Decanoic A	COX4I1
Decanoic A	SCN4A
Decanoic A	CES1
Decanoic A	COX2
Decanoic A	SUCLG2

Decanoic ASLC25A10
Decanoic ASUCNR1
Decanoic AOXCT1
Decanoic ASDHA
Decanoic ADCT
Decanoic AJMJD6
Decanoic AHSD17B11
Decanoic AP4HB
Decanoic AGRIN3B
Decanoic ACACNA1B
Decanoic AGRIN3A
Decanoic AGRIN1
Decanoic ATYR
Decanoic ASCN1A
Decanoic ACOX7C
Decanoic ASCN5A
Decanoic ACOX3
Decanoic AHDAC9
Decanoic ASCN4B
Decanoic AACADSB
Decanoic AOGDH
Decanoic ASLC13A2
Decanoic AP3H3
Decanoic ASLC13A3
Decanoic APLOD3
Decanoic AP4HA2
Decanoic AAKR1C3
Decanoic ASLC13A4
Decanoic ATYRP1
Decanoic ACACNA2D1
Decanoic ACACNA2D2
Decanoic AGRIN2C
Decanoic ASLC7A1
Decanoic ASRD5A2
Decanoic AESRRG
Decanoic ACOX1
Decanoic ASCN10A
Decanoic AAR
Decanoic APLA2G1B
Decanoic AADH1C
Decanoic ASCN8A
Decanoic AHDAC2
Decanoic ASLC13A1
Decanoic AOXCT2
Decanoic AASPH
Decanoic AP4HA1
Decanoic ASUCLA2
Decanoic ACRTAP
Decanoic ASDHAF2
Decanoic AHIF1AN
Decanoic APLAT
Decanoic AGRIN2A
Decanoic AGRIN2B
Decanoic ASLC7A4
Decanoic ASCN11A
Decanoic ASCN3B
Decanoic ASCN3A
Decanoic AALDH5A1

Decanoic A	COX7A1
Decanoic A	COX6A2
Decanoic A	COX6B1
Decanoic A	COX7B
Decanoic A	COX8A
Decanoic A	SUCLG1
Decanoic A	TMLHE
Decanoic A	BBOX1
Decanoic A	SDHC
Decanoic A	SDHD
Decanoic A	SLIT2
Decanoic A	SLC1A3
Decanoic A	UROS
Decanoic A	SLC7A2
Decanoic A	TOP1
Decanoic A	SLC7A3
Decanoic A	GRIN2D
Decanoic A	SCN2B
Decanoic A	COX6C
Decanoic A	AKR1C2
Decanoic A	SCN2A
Decanoic A	FECH
Decanoic A	SCN9A
Decanoic A	FABP6
Decanoic A	SCN1B
Decanoic A	NR1H4
Decanoic A	HSD17B6
Decanoic A	PLOD1
Decanoic A	SDHB
Decanoic A	P3H2
Decanoic A	P3H1
Decanoic A	ACO2
Decanoic A	SALL1
Decanoic A	SLC13A5
Decanoic A	CACNA1A
Decanoic A	PLG
Decanoic A	KARS
Decanoic A	ADORA1
Dihydropir	GABRA2
Dihydropir	TNF
Dihydropir	GABRP
Dihydropir	GABRQ
Dihydropir	PDE4B
Dihydropir	COLQ
Dihydropir	OPRK1
Dihydropir	SLC6A2
Dihydropir	GABRD
Dihydropir	GABRE
Dihydropir	GABRA4
Dihydropir	GABRB3
Dihydropir	BCHE
Dihydropir	LTA
Dihydropir	SLC6A4
Dihydropir	PDE3A
Dihydropir	SCN2A
Dihydropir	GABRB2
Dihydropir	GABRA5
Dihydropir	GABRG2

Dihydropir	RXRA
Dihydropir	OPRM1
Dihydropir	GABRB1
Dihydropir	GABRA3
Dihydropir	ACHE
Dihydropir	GABRA6
Dihydropir	PDE3B
Dihydropir	ADRA1A
Dihydropir	ALOX5
Dihydropir	GABRG3
Dihydropir	GABRG1
Dihydropir	SCN4A
Dihydropir	GABRA1
Dihydropir	PDE4D
Dihydropir	OPRD1
Dihydropir	HTR3A
Bicycloeler	KCND1
Bicycloeler	GAMT
Bicycloeler	CAT
Bicycloeler	KCNC1
Bicycloeler	KCND2
Bicycloeler	KCND3
Bicycloeler	KCNQ1
Bicycloeler	RDH13
Bicycloeler	ESR1
Bicycloeler	RXRG
Bicycloeler	RLBP1
Bicycloeler	LRAT
Bicycloeler	KCNA3
Bicycloeler	KCNC3
Bicycloeler	KCNB1
Bicycloeler	ADH1C
Bicycloeler	ALDH2
Bicycloeler	GUCY1B3
Bicycloeler	RDH11
Bicycloeler	DHRS3
Bicycloeler	RARG
Bicycloeler	RDH12
Bicycloeler	RDH14
Bicycloeler	RXRA
Bicycloeler	PRKAB1
Bicycloeler	KCNA1
Bicycloeler	ADH1B
Bicycloeler	KCNB2
Bicycloeler	KCNA4
Bicycloeler	GATM
Bicycloeler	RXRB
Bicycloeler	RDH5
Bicycloeler	PGR
Bicycloeler	DHRS4
Bicycloeler	RARA
Bicycloeler	ALDH1A2
Bicycloeler	ADH1A
Bicycloeler	KCNA2
Bicycloeler	DLG4
Bicycloeler	KCNA5
Bicycloeler	KCNA7
Bicycloeler	KCNK4

Bicycloeler	RBP3
Bicycloeler	RARRES1
Bicycloeler	RARB
Bicycloeler	RBP1
Bicycloeler	RDH8
Bicycloeler	VDR
Bicycloeler	KCNA10
Bicycloeler	TPO
Bicycloeler	KCNC2
Bicycloeler	RNASE1
Bicycloeler	KCNA6
Bicycloeler	IYD
Bicycloeler	RETSAT
Bicycloeler	NR0B1
Bicycloeler	ALDH1A3
Bicycloeler	ALDH1A1
Bicycloeler	GPRC5A
Alloocimer	KCND1
Alloocimer	GAMT
Alloocimer	CAT
Alloocimer	KCNC1
Alloocimer	KCND2
Alloocimer	KCND3
Alloocimer	KCNQ1
Alloocimer	KCNA3
Alloocimer	KCNC3
Alloocimer	KCNB1
Alloocimer	ADH1C
Alloocimer	ALDH2
Alloocimer	GUCY1B3
Alloocimer	PRKAB1
Alloocimer	KCNA1
Alloocimer	ADH1B
Alloocimer	KCNB2
Alloocimer	KCNA4
Alloocimer	GATM
Alloocimer	ADH1A
Alloocimer	KCNA2
Alloocimer	DLG4
Alloocimer	KCNA5
Alloocimer	KCNA7
Alloocimer	KCNK4
Alloocimer	KCNA10
Alloocimer	TPO
Alloocimer	KCNC2
Alloocimer	RNASE1
Alloocimer	KCNA6
Alloocimer	IYD
1-Methyl-:	HTR4
1-Methyl-:	NQO2
1-Methyl-:	GGCX
1-Methyl-:	OPRM1
1-Methyl-:	HTR1A
1-Methyl-:	AGTR1
1-Methyl-:	AVP
1-Methyl-:	WLS
1-Methyl-:	GABRA2
1-Methyl-:	HTR1B

1-Methyl-:	F10
1-Methyl-:	AGTR2
1-Methyl-:	PTGS2
1-Methyl-:	VKORC1L1
1-Methyl-:	PAX2
1-Methyl-:	GPR27
1-Methyl-:	PROS1
1-Methyl-:	GABRA3
1-Methyl-:	VKORC1
1-Methyl-:	BGLAP
1-Methyl-:	HTR3A
1-Methyl-:	F2
1-Methyl-:	DAB2
1-Methyl-:	SERPINB7
1-Methyl-:	PROZ
1-Methyl-:	F7
1-Methyl-:	JUN
1-Methyl-:	PTGS1
1-Methyl-:	GABRA1
1-Methyl-:	AGT
1-Methyl-:	MGP
1-Methyl-:	ACTN2
1-Methyl-:	AVPR1A
1-Methyl-:	F9
1-Methyl-:	PROC
1-Methyl-:	AVPR2
1-Methyl-:	NQO1
1-Methyl-:	CBR4
1-Methyl-:	F3
Limonene	KCND1
Limonene	GAMT
Limonene	CAT
Limonene	KCNC1
Limonene	KCND2
Limonene	KCND3
Limonene	KCNQ1
Limonene	NR3C2
Limonene	KCNA3
Limonene	KCNC3
Limonene	KCNB1
Limonene	ADH1C
Limonene	ALDH2
Limonene	GUCY1B3
Limonene	CYP17A1
Limonene	PRKAB1
Limonene	KCNA1
Limonene	ADH1B
Limonene	KCNB2
Limonene	KCNA4
Limonene	GATM
Limonene	ESR1
Limonene	ADH1A
Limonene	KCNA2
Limonene	DLG4
Limonene	KCNA5
Limonene	KCNA7
Limonene	KCNK4
Limonene	PGR

Limonene	KCNA10
Limonene	TPO
Limonene	KCNC2
Limonene	RNASE1
Limonene	KCNA6
Limonene	IYD
Limonene	OPRK1
Beta-Bisab	KCND1
Beta-Bisab	GAMT
Beta-Bisab	CAT
Beta-Bisab	KCNC1
Beta-Bisab	KCND2
Beta-Bisab	KCND3
Beta-Bisab	KCNQ1
Beta-Bisab	NR3C2
Beta-Bisab	KCNA3
Beta-Bisab	KCNC3
Beta-Bisab	KCNB1
Beta-Bisab	ADH1C
Beta-Bisab	ALDH2
Beta-Bisab	GUCY1B3
Beta-Bisab	CYP17A1
Beta-Bisab	PRKAB1
Beta-Bisab	KCNA1
Beta-Bisab	ADH1B
Beta-Bisab	KCNB2
Beta-Bisab	KCNA4
Beta-Bisab	GATM
Beta-Bisab	ESR1
Beta-Bisab	ADH1A
Beta-Bisab	KCNA2
Beta-Bisab	DLG4
Beta-Bisab	KCNA5
Beta-Bisab	KCNA7
Beta-Bisab	KCNK4
Beta-Bisab	PGR
Beta-Bisab	KCNA10
Beta-Bisab	TPO
Beta-Bisab	KCNC2
Beta-Bisab	RNASE1
Beta-Bisab	KCNA6
Beta-Bisab	IYD
Beta-Bisab	OPRK1
Alpha-Pine	KCND1
Alpha-Pine	GAMT
Alpha-Pine	CAT
Alpha-Pine	KCNC1
Alpha-Pine	KCND2
Alpha-Pine	KCND3
Alpha-Pine	KCNQ1
Alpha-Pine	RDH13
Alpha-Pine	ALDH1A3
Alpha-Pine	ALDH1A1
Alpha-Pine	ALDH1A2
Alpha-Pine	KCNA3
Alpha-Pine	KCNC3
Alpha-Pine	KCNB1
Alpha-Pine	ADH1C

Alpha-Pine	ALDH2
Alpha-Pine	GUCY1B3
Alpha-Pine	RDH11
Alpha-Pine	DHRS3
Alpha-Pine	RDH12
Alpha-Pine	RLBP1
Alpha-Pine	NR3C2
Alpha-Pine	PRKAB1
Alpha-Pine	KCNA1
Alpha-Pine	ADH1B
Alpha-Pine	KCNB2
Alpha-Pine	KCNA4
Alpha-Pine	GATM
Alpha-Pine	RBP3
Alpha-Pine	RDH5
Alpha-Pine	OPRK1
Alpha-Pine	RDH14
Alpha-Pine	ADH1A
Alpha-Pine	KCNA2
Alpha-Pine	DLG4
Alpha-Pine	KCNA5
Alpha-Pine	KCNA7
Alpha-Pine	KCNK4
Alpha-Pine	RETSAT
Alpha-Pine	ESR1
Alpha-Pine	DHRS4
Alpha-Pine	RDH8
Alpha-Pine	KCNA10
Alpha-Pine	TPO
Alpha-Pine	KCNC2
Alpha-Pine	RNASE1
Alpha-Pine	KCNA6
Alpha-Pine	IYD
Alpha-Pine	CYP17A1
Alpha-Pine	PGR
Alpha-Pine	RBP1
Alpha-Pine	LRAT
Hexadecar	AKR1D1
Hexadecar	ABAT
Hexadecar	COX5B
Hexadecar	SCN7A
Hexadecar	COX5A
Hexadecar	COX4I1
Hexadecar	SCN4A
Hexadecar	CES1
Hexadecar	COX2
Hexadecar	SUCLG2
Hexadecar	SLC25A10
Hexadecar	SUCNR1
Hexadecar	OXCT1
Hexadecar	SDHA
Hexadecar	DCT
Hexadecar	JMJD6
Hexadecar	HSD17B11
Hexadecar	P4HB
Hexadecar	GRIN3B
Hexadecar	CACNA1B
Hexadecar	GRIN3A

Hexadecar	GRIN1
Hexadecar	TYR
Hexadecar	SCN1A
Hexadecar	COX7C
Hexadecar	SCN5A
Hexadecar	COX3
Hexadecar	HDAC9
Hexadecar	SCN4B
Hexadecar	ACADSB
Hexadecar	OGDH
Hexadecar	SLC13A2
Hexadecar	P3H3
Hexadecar	SLC13A3
Hexadecar	PLOD3
Hexadecar	P4HA2
Hexadecar	AKR1C3
Hexadecar	SLC13A4
Hexadecar	TYRP1
Hexadecar	CACNA2D1
Hexadecar	CACNA2D2
Hexadecar	GRIN2C
Hexadecar	SLC7A1
Hexadecar	SRD5A2
Hexadecar	ESRRG
Hexadecar	COX1
Hexadecar	SCN10A
Hexadecar	AR
Hexadecar	PLA2G1B
Hexadecar	ADH1C
Hexadecar	SCN8A
Hexadecar	HDAC2
Hexadecar	SLC13A1
Hexadecar	OXCT2
Hexadecar	ASPH
Hexadecar	P4HA1
Hexadecar	SUCLA2
Hexadecar	CRTAP
Hexadecar	SDHAF2
Hexadecar	HIF1AN
Hexadecar	PLAT
Hexadecar	GRIN2A
Hexadecar	GRIN2B
Hexadecar	SLC7A4
Hexadecar	SCN11A
Hexadecar	SCN3B
Hexadecar	SCN3A
Hexadecar	ALDH5A1
Hexadecar	COX7A1
Hexadecar	COX6A2
Hexadecar	COX6B1
Hexadecar	COX7B
Hexadecar	COX8A
Hexadecar	SUCLG1
Hexadecar	TMLHE
Hexadecar	BBOX1
Hexadecar	SDHC
Hexadecar	SDHD
Hexadecar	SLIT2

Hexadecar	SLC1A3
Hexadecar	UROS
Hexadecar	SLC7A2
Hexadecar	TOP1
Hexadecar	SLC7A3
Hexadecar	GRIN2D
Hexadecar	SCN2B
Hexadecar	COX6C
Hexadecar	AKR1C2
Hexadecar	SCN2A
Hexadecar	FECH
Hexadecar	SCN9A
Hexadecar	FABP6
Hexadecar	SCN1B
Hexadecar	NR1H4
Hexadecar	HSD17B6
Hexadecar	PLOD1
Hexadecar	SDHB
Hexadecar	P3H2
Hexadecar	P3H1
Hexadecar	ACO2
Hexadecar	SALL1
Hexadecar	SLC13A5
Hexadecar	CACNA1A
Hexadecar	PLG
Hexadecar	KARS
Hexadecar	ADORA1
6-Undecar	TRPV3
6-Undecar	SCN11A
6-Undecar	SCN3A
6-Undecar	ALDH5A1
6-Undecar	SCN4A
6-Undecar	SCN1B
6-Undecar	OPRK1
6-Undecar	SCN2B
6-Undecar	SCN7A
6-Undecar	SCN2A
6-Undecar	SCN4B
6-Undecar	OGDH
6-Undecar	TRPM8
6-Undecar	ABAT
6-Undecar	SCN5A
6-Undecar	HDAC9
6-Undecar	SRD5A2
6-Undecar	HDAC2
6-Undecar	TRPA1
6-Undecar	SCN1A
6-Undecar	SCN10A
6-Undecar	TYR
6-Undecar	ACADSB
6-Undecar	KCNK4
6-Undecar	SCN3B
6-Undecar	AKR1D1
6-Undecar	SCN9A
6-Undecar	SCN8A
M-Ethylph	GABRA2
M-Ethylph	TNF
M-Ethylph	GABRP

M-Ethylph	GABRQ
M-Ethylph	PDE4B
M-Ethylph	COLQ
M-Ethylph	SLC6A4
M-Ethylph	DRD3
M-Ethylph	GABRD
M-Ethylph	GABRE
M-Ethylph	GABRA4
M-Ethylph	GABRB3
M-Ethylph	BCHE
M-Ethylph	LTA
M-Ethylph	DRD4
M-Ethylph	ALOX5
M-Ethylph	PDE3A
M-Ethylph	SCN2A
M-Ethylph	GABRB2
M-Ethylph	GABRA5
M-Ethylph	GABRG2
M-Ethylph	RXRA
M-Ethylph	HTR7
M-Ethylph	DRD5
M-Ethylph	GABRB1
M-Ethylph	GABRA3
M-Ethylph	ACHE
M-Ethylph	GABRA6
M-Ethylph	PDE3B
M-Ethylph	SLC6A3
M-Ethylph	HTR1A
M-Ethylph	DRD1
M-Ethylph	GABRG3
M-Ethylph	GABRG1
M-Ethylph	SCN4A
M-Ethylph	GABRA1
M-Ethylph	PDE4D
M-Ethylph	DRD2
M-Ethylph	DBH
M-Ethylph	SLC6A2
Carvacrol /	SCN10A
Carvacrol /	BCHE
Carvacrol /	ACHE
Carvacrol /	COLQ
Alpha-Acc	KCND1
Alpha-Acc	GAMT
Alpha-Acc	CAT
Alpha-Acc	KCNC1
Alpha-Acc	KCND2
Alpha-Acc	KCND3
Alpha-Acc	KCNQ1
Alpha-Acc	NR3C2
Alpha-Acc	KCNA3
Alpha-Acc	KCNC3
Alpha-Acc	KCNB1
Alpha-Acc	ADH1C
Alpha-Acc	ALDH2
Alpha-Acc	GUCY1B3
Alpha-Acc	CYP17A1
Alpha-Acc	PRKAB1
Alpha-Acc	KCNA1

Alpha-Acc	ADH1B
Alpha-Acc	KCNB2
Alpha-Acc	KCNA4
Alpha-Acc	GATM
Alpha-Acc	ESR1
Alpha-Acc	ADH1A
Alpha-Acc	KCNA2
Alpha-Acc	DLG4
Alpha-Acc	KCNA5
Alpha-Acc	KCNA7
Alpha-Acc	KCNK4
Alpha-Acc	PGR
Alpha-Acc	KCNA10
Alpha-Acc	TPO
Alpha-Acc	KCNC2
Alpha-Acc	RNASE1
Alpha-Acc	KCNA6
Alpha-Acc	IYD
Alpha-Acc	OPRK1
Adenine	ADORA2A
Adenine	PRKAB1
Adenine	ADCY1
Adenine	ADK
Adenine	RRM2
Adenine	PIM1
Adenine	AMD1
Adenine	RRM1
Adenine	ADORA2B
Adenine	ACSS2
Adenine	COMT
Adenine	GNMT
Adenine	PRKAA1
Adenine	PYGL
Adenine	ACSL1
Adenine	ADORA3
Adenine	POLE
Adenine	POLA1
Adenine	PNP
Adenine	MAT1A
Adenine	POLE2
Adenine	PDE4B
Adenine	ADORA1
Adenine	CBS
Adenine	DCK
Adenine	PDE4D
Adenine	CREB1
Adenine	HINT1
Adenine	PRKAB2
Adenine	FBP1
Adenine	POLE3
Adenine	RRM2B
Adenine	CBSL
Adenine	ACSS1
Adenine	MAT2A
Adenine	POLE4
Isococculic	CHRM3
Isococculic	CHRM2
Isococculic	DRD2

Isococculic	SLC18A2
Isococculic	CHRNA2
Isococculic	BCHE
O-Cresol	GABRA2
O-Cresol	TNF
O-Cresol	GABRP
O-Cresol	GABRQ
O-Cresol	PDE4B
O-Cresol	COLQ
O-Cresol	GABRD
O-Cresol	GABRE
O-Cresol	GABRA4
O-Cresol	GABRB3
O-Cresol	BCHE
O-Cresol	LTA
O-Cresol	PDE3A
O-Cresol	SCN2A
O-Cresol	GABRB2
O-Cresol	GABRA5
O-Cresol	GABRG2
O-Cresol	RXRA
O-Cresol	GABRB1
O-Cresol	GABRA3
O-Cresol	ACHE
O-Cresol	GABRA6
O-Cresol	PDE3B
O-Cresol	GABRG3
O-Cresol	GABRG1
O-Cresol	SCN4A
O-Cresol	GABRA1
O-Cresol	PDE4D
Retinol	RDH11
Retinol	RDH5
Retinol	ALDH1A1
Retinol	ALDH1A2
Retinol	RARRES1
Retinol	RARB
Retinol	VDR
Retinol	RBP3
Retinol	ALDH1A3
Retinol	RLBP1
Retinol	ALDH3A1
Retinol	NR0B1
Retinol	RXRG
Retinol	CYP27B1
Retinol	RETSAT
Retinol	RDH12
Retinol	RDH14
Retinol	RHO
Retinol	ESR1
Retinol	RARA
Retinol	RDH13
Retinol	DHRS4
Retinol	RDH8
Retinol	RS1
Retinol	RARG
Retinol	GPRC5A
Retinol	DHRS3

Retinol	RBP1
Retinol	LRAT
Retinol	RXRB
Retinol	PGR
Retinol	RXRA
1,2-Benzer	PLAT
1,2-Benzer	BCL2
1,2-Benzer	PTGS2
1,2-Benzer	IL13
1,2-Benzer	AKR1C1
1,2-Benzer	HCAR2
1,2-Benzer	FABP2
1,2-Benzer	NNMT
1,2-Benzer	THBD
1,2-Benzer	CFTR
1,2-Benzer	APOA2
1,2-Benzer	KCNE2
1,2-Benzer	PPARG
1,2-Benzer	HCAR3
1,2-Benzer	TCAF1
1,2-Benzer	ANXA1
1,2-Benzer	QPRT
1,2-Benzer	PTGS1
1,2-Benzer	IL1B
1,2-Benzer	SERPINB7
Dimethyl C	NPR1
Dimethyl C	COX1
Dimethyl C	AR
Dimethyl C	COX6A2
Dimethyl C	CES1
Dimethyl C	NR1H4
Dimethyl C	ESRRG
Dimethyl C	AKR1C2
Dimethyl C	COX7A1
Dimethyl C	TYR
Dimethyl C	SRD5A2
Dimethyl C	COX6C
Dimethyl C	AKR1D1
Dimethyl C	FECH
Dimethyl C	ADH1C
Dimethyl C	COX7B
Dimethyl C	COX5B
Dimethyl C	COX5A
Dimethyl C	COX4I1
Dimethyl C	COX6B1
Dimethyl C	COX2
Dimethyl C	COX7C
Dimethyl C	COX3
Dimethyl C	PLA2G1B
Dimethyl C	FABP6
Dimethyl C	COX8A
Cedrol	NPR1
Cedrol	COX1
Cedrol	AR
Cedrol	COX6A2
Cedrol	CES1
Cedrol	NR1H4
Cedrol	ESRRG

Cedrol	AKR1C2
Cedrol	COX7A1
Cedrol	TYR
Cedrol	SRD5A2
Cedrol	COX6C
Cedrol	AKR1D1
Cedrol	FECH
Cedrol	ADH1C
Cedrol	COX7B
Cedrol	COX5B
Cedrol	COX5A
Cedrol	COX4I1
Cedrol	COX6B1
Cedrol	COX2
Cedrol	COX7C
Cedrol	COX3
Cedrol	PLA2G1B
Cedrol	FABP6
Cedrol	COX8A
Choline	PLD1
Choline	PLD2
Choline	PCYT1A
Choline	BCHE
Choline	PCYT1B
Choline	COLQ
Choline	PHOSPHO1
Choline	ACHE
3(S)-3-But	CHRM3
3(S)-3-But	AR
3(S)-3-But	CHRM1
3(S)-3-But	NR3C1
3(S)-3-But	CHRM2
3(S)-3-But	CYP19A1
3(S)-3-But	ESR1
3(S)-3-But	NR3C2
3(S)-3-But	PGR
1-Tetradec	TRPV3
1-Tetradec	GRIA4
1-Tetradec	CHRNB3
1-Tetradec	GABRG3
1-Tetradec	GABRA3
1-Tetradec	CHRNA10
1-Tetradec	CACNA1C
1-Tetradec	CHRNA2
1-Tetradec	HTR3B
1-Tetradec	GABRA6
1-Tetradec	CHRNA5
1-Tetradec	CACNG1
1-Tetradec	DMTN
1-Tetradec	SCN1A
1-Tetradec	SCN10A
1-Tetradec	TYR
1-Tetradec	ACADSB
1-Tetradec	OPRK1
1-Tetradec	GABRA2
1-Tetradec	GABRB1
1-Tetradec	VCAM1
1-Tetradec	SLC29A1

1-Tetradec	GRIA2
1-Tetradec	CHRNB2
1-Tetradec	CHRNA6
1-Tetradec	GRIA3
1-Tetradec	HTR3A
1-Tetradec	CHRNB4
1-Tetradec	ADAM8
1-Tetradec	KCNMA1
1-Tetradec	SCN3B
1-Tetradec	AKR1D1
1-Tetradec	SCN9A
1-Tetradec	SCN8A
1-Tetradec	TRPM8
1-Tetradec	CACNG2
1-Tetradec	GRIA1
1-Tetradec	CHRNA7
1-Tetradec	CACNA1S
1-Tetradec	GABRA4
1-Tetradec	CACNA1D
1-Tetradec	GABRB3
1-Tetradec	GABRA5
1-Tetradec	GABRA1
1-Tetradec	KCNJ9
1-Tetradec	SLC28A3
1-Tetradec	SCN11A
1-Tetradec	SCN3A
1-Tetradec	ALDH5A1
1-Tetradec	SCN4A
1-Tetradec	SCN1B
1-Tetradec	TRPA1
1-Tetradec	GABRD
1-Tetradec	GLRA2
1-Tetradec	KCNJ6
1-Tetradec	GABRG1
1-Tetradec	CHRNA3
1-Tetradec	HTR3D
1-Tetradec	KCNJ5
1-Tetradec	SLC29A2
1-Tetradec	CHRNA9
1-Tetradec	HTR3C
1-Tetradec	ADRA1B
1-Tetradec	SCN2B
1-Tetradec	SCN7A
1-Tetradec	SCN2A
1-Tetradec	SCN4B
1-Tetradec	OGDH
1-Tetradec	CACNB1
1-Tetradec	CHRNA4
1-Tetradec	KCNJ3
1-Tetradec	GABRE
1-Tetradec	GABRP
1-Tetradec	GABRB2
1-Tetradec	GABRQ
1-Tetradec	GRIN3A
1-Tetradec	HTR3E
1-Tetradec	CHRFAM7A
1-Tetradec	GLRA1
1-Tetradec	FOXL2

1-Tetradec	ABAT
1-Tetradec	SCN5A
1-Tetradec	HDAC9
1-Tetradec	SRD5A2
1-Tetradec	HDAC2
Anisic Acid	RXRB
Anisic Acid	PTGS2
Anisic Acid	TNF
Anisic Acid	PRKAG2
Anisic Acid	HSPA5
Anisic Acid	PRKAG3
Anisic Acid	RARG
Anisic Acid	RARA
Anisic Acid	HNRNPK
Anisic Acid	NFKB1
Anisic Acid	PRKAA2
Anisic Acid	IKBKB
Anisic Acid	RARB
Anisic Acid	PPARA
Anisic Acid	COL27A1
Anisic Acid	EDNRA
Anisic Acid	AKR1C1
Anisic Acid	PRKAB2
Anisic Acid	RXRG
Anisic Acid	RXRA
Anisic Acid	SERPINB7
Anisic Acid	NFKBIA
Anisic Acid	PRKAG1
Anisic Acid	RPS6KA3
Anisic Acid	PTGS1
Anisic Acid	TP53
Anisic Acid	PRKAB1
Anisic Acid	NFKB2
Anisic Acid	PRKAA1
Phyllanthir	ESR2
Phyllanthir	ESR1
Phyllanthir	NR1I2
Phyllanthir	ALOX5
Pentylbenz	GABRB2
Pentylbenz	ADRA1A
Pentylbenz	ADRB1
Pentylbenz	SLC18A2
Pentylbenz	TAAR1
Pentylbenz	GABRB3
Pentylbenz	S1PR5
Pentylbenz	ADRA1D
Pentylbenz	ADRA1B
Pentylbenz	CARTPT
Pentylbenz	F2
Pentylbenz	ADRB2
Pentylbenz	DRD2
Pentylbenz	ADRA2C
Pentylbenz	ADRA2B
Pentylbenz	GPR27
Pentylbenz	ADRA2A
Pentylbenz	MAOA
Pentylbenz	ALOX5
Pentylbenz	SLC6A2

Pentylbenz	SLC6A3
Pentylbenz	MAOB
Pentylbenz	SLC6A4
Pentylbenz	ADRB3
Camphene	KCND1
Camphene	GAMT
Camphene	CAT
Camphene	KCNC1
Camphene	KCND2
Camphene	KCND3
Camphene	KCNQ1
Camphene	KCNA3
Camphene	KCNC3
Camphene	KCNB1
Camphene	ADH1C
Camphene	ALDH2
Camphene	GUCY1B3
Camphene	PRKAB1
Camphene	KCNA1
Camphene	ADH1B
Camphene	KCNB2
Camphene	KCNA4
Camphene	GATM
Camphene	ADH1A
Camphene	KCNA2
Camphene	DLG4
Camphene	KCNA5
Camphene	KCNA7
Camphene	KCNK4
Camphene	KCNA10
Camphene	TPO
Camphene	KCNC2
Camphene	RNASE1
Camphene	KCNA6
Camphene	IYD
20-Hexade	PRKCA
20-Hexade	PRKCD
P-Cresol	GABRA2
P-Cresol	TNF
P-Cresol	GABRP
P-Cresol	GABRQ
P-Cresol	PDE4B
P-Cresol	COLQ
P-Cresol	GABRD
P-Cresol	GABRE
P-Cresol	GABRA4
P-Cresol	GABRB3
P-Cresol	BCHE
P-Cresol	LTA
P-Cresol	PDE3A
P-Cresol	SCN2A
P-Cresol	GABRB2
P-Cresol	GABRA5
P-Cresol	GABRG2
P-Cresol	RXRA
P-Cresol	GABRB1
P-Cresol	GABRA3
P-Cresol	ACHE

P-Cresol	GABRA6
P-Cresol	PDE3B
P-Cresol	GABRG3
P-Cresol	GABRG1
P-Cresol	SCN4A
P-Cresol	GABRA1
P-Cresol	PDE4D
Vitamin B1	SLC19A2
Vitamin B1	TPK1
Vitamin B1	THTPA
1,2-Dimetl	KCND1
1,2-Dimetl	GAMT
1,2-Dimetl	CAT
1,2-Dimetl	KCNC1
1,2-Dimetl	KCND2
1,2-Dimetl	KCND3
1,2-Dimetl	KCNQ1
1,2-Dimetl	KCNA3
1,2-Dimetl	KCNC3
1,2-Dimetl	KCNB1
1,2-Dimetl	ADH1C
1,2-Dimetl	ALDH2
1,2-Dimetl	GUCY1B3
1,2-Dimetl	PRKAB1
1,2-Dimetl	KCNA1
1,2-Dimetl	ADH1B
1,2-Dimetl	KCNB2
1,2-Dimetl	KCNA4
1,2-Dimetl	GATM
1,2-Dimetl	ADH1A
1,2-Dimetl	KCNA2
1,2-Dimetl	DLG4
1,2-Dimetl	KCNA5
1,2-Dimetl	KCNA7
1,2-Dimetl	KCNK4
1,2-Dimetl	KCNA10
1,2-Dimetl	TPO
1,2-Dimetl	KCNC2
1,2-Dimetl	RNASE1
1,2-Dimetl	KCNA6
1,2-Dimetl	IYD
Cnidilide	CHRM3
Cnidilide	AR
Cnidilide	HDAC2
Cnidilide	CHRM1
Cnidilide	NR3C1
Cnidilide	NR3C2
Cnidilide	CHRM2
Cnidilide	HMGCR
Cnidilide	ESR1
Cnidilide	ITGB2
Cnidilide	PGR
Cnidilide	ITGAL
Beta-Acora	KCND1
Beta-Acora	GAMT
Beta-Acora	CAT
Beta-Acora	KCNC1
Beta-Acora	KCND2

Beta-Acora	KCND3
Beta-Acora	KCNQ1
Beta-Acora	NR3C2
Beta-Acora	KCNA3
Beta-Acora	KCNC3
Beta-Acora	KCNB1
Beta-Acora	ADH1C
Beta-Acora	ALDH2
Beta-Acora	GUCY1B3
Beta-Acora	CYP17A1
Beta-Acora	PRKAB1
Beta-Acora	KCNA1
Beta-Acora	ADH1B
Beta-Acora	KCNB2
Beta-Acora	KCNA4
Beta-Acora	GATM
Beta-Acora	ESR1
Beta-Acora	ADH1A
Beta-Acora	KCNA2
Beta-Acora	DLG4
Beta-Acora	KCNA5
Beta-Acora	KCNA7
Beta-Acora	KCNK4
Beta-Acora	PGR
Beta-Acora	KCNA10
Beta-Acora	TPO
Beta-Acora	KCNC2
Beta-Acora	RNASE1
Beta-Acora	KCNA6
Beta-Acora	IYD
Beta-Acora	OPRK1
Decanal	SCN11A
Decanal	SCN3A
Decanal	ALDH5A1
Decanal	SCN4A
Decanal	SCN1B
Decanal	SCN2B
Decanal	SCN7A
Decanal	SCN2A
Decanal	SCN4B
Decanal	OGDH
Decanal	ABAT
Decanal	SCN5A
Decanal	HDAC9
Decanal	SRD5A2
Decanal	HDAC2
Decanal	SCN1A
Decanal	SCN10A
Decanal	TYR
Decanal	ACADSB
Decanal	SCN3B
Decanal	AKR1D1
Decanal	SCN9A
Decanal	SCN8A
Brefeldin A	HMGCR
Brefeldin A	PGR
Brefeldin A	ITGB2
Brefeldin A	AR

Brefeldin A	ITGAL
Brefeldin A	HDAC2
Brefeldin A	ESR1
Stigmaster	KCND1
Stigmaster	GAMT
Stigmaster	CAT
Stigmaster	KCNC1
Stigmaster	KCND2
Stigmaster	KCND3
Stigmaster	KCNQ1
Stigmaster	RDH13
Stigmaster	ALDH1A3
Stigmaster	ALDH1A1
Stigmaster	ALDH1A2
Stigmaster	KCNA3
Stigmaster	KCNC3
Stigmaster	KCNB1
Stigmaster	ADH1C
Stigmaster	ALDH2
Stigmaster	GUCY1B3
Stigmaster	RDH11
Stigmaster	DHRS3
Stigmaster	RDH12
Stigmaster	RLBP1
Stigmaster	NR3C2
Stigmaster	PRKAB1
Stigmaster	KCNA1
Stigmaster	ADH1B
Stigmaster	KCNB2
Stigmaster	KCNA4
Stigmaster	GATM
Stigmaster	RBP3
Stigmaster	RDH5
Stigmaster	OPRK1
Stigmaster	RDH14
Stigmaster	ADH1A
Stigmaster	KCNA2
Stigmaster	DLG4
Stigmaster	KCNA5
Stigmaster	KCNA7
Stigmaster	KCNK4
Stigmaster	RETSAT
Stigmaster	ESR1
Stigmaster	DHRS4
Stigmaster	RDH8
Stigmaster	KCNA10
Stigmaster	TPO
Stigmaster	KCNC2
Stigmaster	RNASE1
Stigmaster	KCNA6
Stigmaster	IYD
Stigmaster	CYP17A1
Stigmaster	PGR
Stigmaster	RBP1
Stigmaster	LRAT
Nonanal	SCN11A
Nonanal	SCN3A
Nonanal	ALDH5A1

Nonanal	SCN4A
Nonanal	SCN1B
Nonanal	SCN2B
Nonanal	SCN7A
Nonanal	SCN2A
Nonanal	SCN4B
Nonanal	OGDH
Nonanal	ABAT
Nonanal	SCN5A
Nonanal	HDAC9
Nonanal	SRD5A2
Nonanal	HDAC2
Nonanal	SCN1A
Nonanal	SCN10A
Nonanal	TYR
Nonanal	ACADSB
Nonanal	SCN3B
Nonanal	AKR1D1
Nonanal	SCN9A
Nonanal	SCN8A
Vitamin B1	MUT
Vitamin B1	MTHFR
Vitamin B1	CBS
Vitamin B1	MTRR
Vitamin B1	TCN1
Vitamin B1	CEBPA
Vitamin B1	MTR
Vitamin B1	MMAB
Vitamin B1	WDHD1
Vitamin B1	MMACHC
Vitamin B1	CUBN
Vitamin B1	MMAA
Vitamin B1	AMN
1-Hexadec	TRPV3
1-Hexadec	GRIA4
1-Hexadec	CHRNB3
1-Hexadec	GABRG3
1-Hexadec	GABRA3
1-Hexadec	CHRNA10
1-Hexadec	CACNA1C
1-Hexadec	CHRNA2
1-Hexadec	HTR3B
1-Hexadec	GABRA6
1-Hexadec	CHRNA5
1-Hexadec	CACNG1
1-Hexadec	DMTN
1-Hexadec	SCN1A
1-Hexadec	SCN10A
1-Hexadec	TYR
1-Hexadec	ACADSB
1-Hexadec	OPRK1
1-Hexadec	GABRA2
1-Hexadec	GABRB1
1-Hexadec	VCAM1
1-Hexadec	SLC29A1
1-Hexadec	GRIA2
1-Hexadec	CHRNB2
1-Hexadec	CHRNA6

1-Hexadec	GRIA3
1-Hexadec	HTR3A
1-Hexadec	CHRNB4
1-Hexadec	ADAM8
1-Hexadec	KCNMA1
1-Hexadec	SCN3B
1-Hexadec	AKR1D1
1-Hexadec	SCN9A
1-Hexadec	SCN8A
1-Hexadec	TRPM8
1-Hexadec	CACNG2
1-Hexadec	GRIA1
1-Hexadec	CHRNA7
1-Hexadec	CACNA1S
1-Hexadec	GABRA4
1-Hexadec	CACNA1D
1-Hexadec	GABRB3
1-Hexadec	GABRA5
1-Hexadec	GABRA1
1-Hexadec	KCNJ9
1-Hexadec	SLC28A3
1-Hexadec	SCN11A
1-Hexadec	SCN3A
1-Hexadec	ALDH5A1
1-Hexadec	SCN4A
1-Hexadec	SCN1B
1-Hexadec	TRPA1
1-Hexadec	GABRD
1-Hexadec	GLRA2
1-Hexadec	KCNJ6
1-Hexadec	GABRG1
1-Hexadec	CHRNA3
1-Hexadec	HTR3D
1-Hexadec	KCNJ5
1-Hexadec	SLC29A2
1-Hexadec	CHRNA9
1-Hexadec	HTR3C
1-Hexadec	ADRA1B
1-Hexadec	SCN2B
1-Hexadec	SCN7A
1-Hexadec	SCN2A
1-Hexadec	SCN4B
1-Hexadec	OGDH
1-Hexadec	CACNB1
1-Hexadec	CHRNA4
1-Hexadec	KCNJ3
1-Hexadec	GABRE
1-Hexadec	GABRP
1-Hexadec	GABRB2
1-Hexadec	GABRQ
1-Hexadec	GRIN3A
1-Hexadec	HTR3E
1-Hexadec	CHRFAM7A
1-Hexadec	GLRA1
1-Hexadec	FOXL2
1-Hexadec	ABAT
1-Hexadec	SCN5A
1-Hexadec	HDAC9

1-Hexadec	SRD5A2
1-Hexadec	HDAC2
4-Octanor	SCN3A
4-Octanor	SCN2A
4-Octanor	ACADSB
4-Octanor	SCN11A
4-Octanor	SCN7A
4-Octanor	HDAC9
4-Octanor	SCN8A
4-Octanor	SCN2B
4-Octanor	SCN5A
4-Octanor	SCN9A
4-Octanor	SCN1B
4-Octanor	ABAT
4-Octanor	SCN10A
4-Octanor	SCN4A
4-Octanor	OGDH
4-Octanor	SCN1A
4-Octanor	ALDH5A1
4-Octanor	SCN4B
4-Octanor	HDAC2
P-Ethylphe	GABRA2
P-Ethylphe	TNF
P-Ethylphe	GABRP
P-Ethylphe	GABRQ
P-Ethylphe	PDE4B
P-Ethylphe	COLQ
P-Ethylphe	SLC6A4
P-Ethylphe	DRD3
P-Ethylphe	GABRD
P-Ethylphe	GABRE
P-Ethylphe	GABRA4
P-Ethylphe	GABRB3
P-Ethylphe	BCHE
P-Ethylphe	LTA
P-Ethylphe	DRD4
P-Ethylphe	ALOX5
P-Ethylphe	PDE3A
P-Ethylphe	SCN2A
P-Ethylphe	GABRB2
P-Ethylphe	GABRA5
P-Ethylphe	GABRG2
P-Ethylphe	RXRA
P-Ethylphe	HTR7
P-Ethylphe	DRD5
P-Ethylphe	GABRB1
P-Ethylphe	GABRA3
P-Ethylphe	ACHE
P-Ethylphe	GABRA6
P-Ethylphe	PDE3B
P-Ethylphe	SLC6A3
P-Ethylphe	HTR1A
P-Ethylphe	DRD1
P-Ethylphe	GABRG3
P-Ethylphe	GABRG1
P-Ethylphe	SCN4A
P-Ethylphe	GABRA1
P-Ethylphe	PDE4D

P-Ethylphe	DRD2
P-Ethylphe	DBH
P-Ethylphe	SLC6A2
Camphere	VDR
Camphere	ESR1
Camphere	CYP27B1
Camphere	PGR
Camphere	GC
Camphere	SNW1
Camphere	F12
Beta-Caryo	KCND1
Beta-Caryo	GAMT
Beta-Caryo	CAT
Beta-Caryo	KCNC1
Beta-Caryo	KCND2
Beta-Caryo	KCND3
Beta-Caryo	KCNQ1
Beta-Caryo	KCNA3
Beta-Caryo	KCNC3
Beta-Caryo	KCNB1
Beta-Caryo	ADH1C
Beta-Caryo	ALDH2
Beta-Caryo	GUCY1B3
Beta-Caryo	PRKAB1
Beta-Caryo	KCNA1
Beta-Caryo	ADH1B
Beta-Caryo	KCNB2
Beta-Caryo	KCNA4
Beta-Caryo	GATM
Beta-Caryo	ADH1A
Beta-Caryo	KCNA2
Beta-Caryo	DLG4
Beta-Caryo	KCNA5
Beta-Caryo	KCNA7
Beta-Caryo	KCNK4
Beta-Caryo	KCNA10
Beta-Caryo	TPO
Beta-Caryo	KCNC2
Beta-Caryo	RNASE1
Beta-Caryo	KCNA6
Beta-Caryo	IYD
1-Dodecen	KCND1
1-Dodecen	GAMT
1-Dodecen	CAT
1-Dodecen	KCNC1
1-Dodecen	KCND2
1-Dodecen	KCND3
1-Dodecen	KCNQ1
1-Dodecen	KCNA3
1-Dodecen	KCNC3
1-Dodecen	KCNB1
1-Dodecen	ADH1C
1-Dodecen	ALDH2
1-Dodecen	GUCY1B3
1-Dodecen	PRKAB1
1-Dodecen	KCNA1
1-Dodecen	ADH1B
1-Dodecen	KCNB2

1-Dodecer	KCNA4
1-Dodecer	GATM
1-Dodecer	ADH1A
1-Dodecer	KCNA2
1-Dodecer	DLG4
1-Dodecer	KCNA5
1-Dodecer	KCNA7
1-Dodecer	KCNK4
1-Dodecer	KCNA10
1-Dodecer	TPO
1-Dodecer	KCNC2
1-Dodecer	RNASE1
1-Dodecer	KCNA6
1-Dodecer	IYD
Guaiacol	TYR
Guaiacol	DCT
Guaiacol	TYRP1
M-Cresol	TNF
M-Cresol	GABRP
M-Cresol	GABRQ
M-Cresol	PDE4B
M-Cresol	COLQ
M-Cresol	GABRA2
M-Cresol	GABRE
M-Cresol	GABRA4
M-Cresol	GABRB3
M-Cresol	BCHE
M-Cresol	LTA
M-Cresol	GABRD
M-Cresol	SCN2A
M-Cresol	GABRB2
M-Cresol	GABRA5
M-Cresol	GABRG2
M-Cresol	RXRA
M-Cresol	PDE3A
M-Cresol	GABRA3
M-Cresol	ACHE
M-Cresol	GABRA6
M-Cresol	PDE3B
M-Cresol	GABRB1
M-Cresol	GABRG1
M-Cresol	SCN4A
M-Cresol	GABRA1
M-Cresol	PDE4D
Isofernene	KCND1
Isofernene	GAMT
Isofernene	CAT
Isofernene	KCNC1
Isofernene	KCND2
Isofernene	KCND3
Isofernene	KCNQ1
Isofernene	RDH13
Isofernene	ALDH1A3
Isofernene	ALDH1A1
Isofernene	ALDH1A2
Isofernene	KCNA3
Isofernene	KCNC3
Isofernene	KCNB1

Isofernene	ADH1C
Isofernene	ALDH2
Isofernene	GUCY1B3
Isofernene	RDH11
Isofernene	DHRS3
Isofernene	RDH12
Isofernene	RLBP1
Isofernene	NR3C2
Isofernene	PRKAB1
Isofernene	KCNA1
Isofernene	ADH1B
Isofernene	KCNB2
Isofernene	KCNA4
Isofernene	GATM
Isofernene	RBP3
Isofernene	RDH5
Isofernene	OPRK1
Isofernene	RDH14
Isofernene	ADH1A
Isofernene	KCNA2
Isofernene	DLG4
Isofernene	KCNA5
Isofernene	KCNA7
Isofernene	KCNK4
Isofernene	RETSAT
Isofernene	ESR1
Isofernene	DHRS4
Isofernene	RDH8
Isofernene	KCNA10
Isofernene	TPO
Isofernene	KCNC2
Isofernene	RNASE1
Isofernene	KCNA6
Isofernene	IYD
Isofernene	CYP17A1
Isofernene	PGR
Isofernene	RBP1
Isofernene	LRAT
Hexanoic A	CHRM1
Hexanoic A	CHRM2
Crinamine	CHRNG
Crinamine	CHRNA10
Crinamine	CHRNA2
Crinamine	CHRFAM7A
Crinamine	CHRNA4
Crinamine	CHRNA3
Crinamine	CHRNA6
Crinamine	CHRNA5
Crinamine	CHRNB3
Crinamine	CHRNB2
Crinamine	CHRND
Crinamine	CHRNB4
Crinamine	CHRNA7
Crinamine	ACHE
Crinamine	BCHE
Crinamine	CHRNB1
Crinamine	CHRNA1
Crinamine	CHRNE

Crinamine	CHRNA9
6-Undecar	SCN11A
6-Undecar	SCN3A
6-Undecar	ALDH5A1
6-Undecar	SCN4A
6-Undecar	SCN1B
6-Undecar	SCN2B
6-Undecar	SCN7A
6-Undecar	SCN2A
6-Undecar	SCN4B
6-Undecar	OGDH
6-Undecar	ABAT
6-Undecar	SCN5A
6-Undecar	HDAC9
6-Undecar	SRD5A2
6-Undecar	HDAC2
6-Undecar	SCN1A
6-Undecar	SCN10A
6-Undecar	TYR
6-Undecar	ACADSB
6-Undecar	SCN3B
6-Undecar	AKR1D1
6-Undecar	SCN9A
6-Undecar	SCN8A
Alpha-Cha	KCND1
Alpha-Cha	GAMT
Alpha-Cha	CAT
Alpha-Cha	KCNC1
Alpha-Cha	KCND2
Alpha-Cha	KCND3
Alpha-Cha	KCNQ1
Alpha-Cha	RDH13
Alpha-Cha	ALDH1A3
Alpha-Cha	ALDH1A1
Alpha-Cha	ALDH1A2
Alpha-Cha	KCNA3
Alpha-Cha	KCNC3
Alpha-Cha	KCNB1
Alpha-Cha	ADH1C
Alpha-Cha	ALDH2
Alpha-Cha	GUCY1B3
Alpha-Cha	RDH11
Alpha-Cha	DHRS3
Alpha-Cha	RDH12
Alpha-Cha	RLBP1
Alpha-Cha	NR3C2
Alpha-Cha	PRKAB1
Alpha-Cha	KCNA1
Alpha-Cha	ADH1B
Alpha-Cha	KCNB2
Alpha-Cha	KCNA4
Alpha-Cha	GATM
Alpha-Cha	RBP3
Alpha-Cha	RDH5
Alpha-Cha	OPRK1
Alpha-Cha	RDH14
Alpha-Cha	ADH1A
Alpha-Cha	KCNA2

Alpha-Cha	DLG4
Alpha-Cha	KCNA5
Alpha-Cha	KCNA7
Alpha-Cha	KCNK4
Alpha-Cha	RETSAT
Alpha-Cha	ESR1
Alpha-Cha	DHRS4
Alpha-Cha	RDH8
Alpha-Cha	KCNA10
Alpha-Cha	TPO
Alpha-Cha	KCNC2
Alpha-Cha	RNASE1
Alpha-Cha	KCNA6
Alpha-Cha	IYD
Alpha-Cha	CYP17A1
Alpha-Cha	PGR
Alpha-Cha	RBP1
Alpha-Cha	LRAT
3-Carene	KCND1
3-Carene	GAMT
3-Carene	CAT
3-Carene	KCNC1
3-Carene	KCND2
3-Carene	KCND3
3-Carene	KCNQ1
3-Carene	KCNA3
3-Carene	KCNC3
3-Carene	KCNB1
3-Carene	ADH1C
3-Carene	ALDH2
3-Carene	GUCY1B3
3-Carene	ESR1
3-Carene	PRKAB1
3-Carene	KCNA1
3-Carene	ADH1B
3-Carene	KCNB2
3-Carene	KCNA4
3-Carene	GATM
3-Carene	PGR
3-Carene	ADH1A
3-Carene	KCNA2
3-Carene	DLG4
3-Carene	KCNA5
3-Carene	KCNA7
3-Carene	KCNK4
3-Carene	KCNA10
3-Carene	TPO
3-Carene	KCNC2
3-Carene	RNASE1
3-Carene	KCNA6
3-Carene	IYD
