# Supplementary material for: Network Pharmacology-Based Strategy to Investigate Pharmacological Mechanisms of the Drug Pair Astragalus-Angelica for Treatment of Male Infertility
Source: Evid Based Complement Alternat Med. 2021 Oct 16;2021:8281506. doi: 10.1155/2021/8281506 (PMC8541871; doi:10.1155/2021/8281506)
Supplement: Supplementary Materials — Table S1: available ingredient and target information of Astragalus collected in TCMSP database. Table S2: available ingredient and target information of Astragalus collected in BATMAN-TCM database. Table S3: available ingredient and target information of Angelica collected in TCMSP database. Table S4: available ingredient and target information of Angelica collected in BATMAN-TCM database. Table S5: The drug pair-component-node data of target-disease regulation network. [file 8281506.f1.zip › 8281506.f1/Table S5.The drug pair-component-Node data oftarget-disease regulation network.pdf]

SUID	Degree	name
73	80	quercetin
76	2	Uracil
78	16	Î’ etaine
80	46	isorhamnetin
82	92	kaempferol
84	6	Chrysanthemaxanthin
86	48	Uridine
88	78	Canavanine
90	2	Astragaloside Vi
92	2	Astramembrannin I
94	2	Astragaloside V
96	2	Astragaloside Iii
98	30	Sucrose
100	2	Astragaloside Vii
102	16	Jaranol
105	24	3,9-di-O-methylnissolin
107	50	7-O-methylisomucronulatol
109	56	formononetin
111	36	Calycosin
115	8	Bifendate
117	58	3,5-Dimethoxystilbene
119	46	Guanosine
121	114	Cetylic Acid
123	56	Myristic Acid
125	56	Lignoceric Acid
127	46	Dodecenoic Acid
129	70	Sebiferic Acid
132	28	Phenylacetic Acid
134	20	Pentylbenzene
136	88	Stigmasterol
138	16	FERULIC ACID (CIS)
140	20	vanillin
145	4	3-O-trans ferulylquinic acid
147	2	7-Hydroxycoumarin
149	8	Scopoletol
151	42	Kumatakenin
154	24	1,2-Benzenedicarboxylic Acid
156	84	beta-sitosterol
161	40	Carvacrol
163	34	4-Ethylresorcinol
165	34	M-Ethylphenol
167	34	P-Ethylphenol
172	38	1-Methyl-2-Dodecyl-4-(1h)-Quinolone
174	6	Dimethyl Phthalate
180	24	Betaine
190	20	Ethanol
192	26	1-Tetradecanol
194	26	1-Hexadecanol
196	12	3(S)-3-Butyl-4,5-Dihydrophthalide
203	14	Succinic Acid

205	14	Sebacic Acid
207	62	Azelaic Acid
210	22	Dimethyl-Beta-Propiothetin
212	48	Decanoic Acid
214	48	Hexadecanoic Acid
216	24	4-Hydroxycoumarin,Folinic Acid
219	16	3-Hydroxycoumarin,Folic Acid
222	50	Adenine
224	12	Vitamin B12
227	38	Anisic Acid
229	22	Choline
232	16	Cnidilide
234	14	Brefeldin A
243	38	Angelicin
250	40	Retinol
286	10	20-Hexadecanoylingenol
302	8	Crinamine
323	2	FA
327	18	24,24-Dimethyl-5alpha-Cholesta-8-En-3beta-Ol
329	14	Alpha-Terpineol
332	40	Bicycloelemene
334	20	Limonene
336	20	Beta-Bisabolene
338	32	Alpha-Pinene
340	20	Alpha-Acoradiene
345	20	Beta-Acoradiene
349	10	Campherenol
351	32	Isofernene
353	32	Alpha-Chamigrene
355	18	3-Carene
359	30	itosterol,Î’ -Sitosterol
361	2	Mairin
363	16	hederagenin
366	10	Soyasapogenol B
369	40	Gamma-Sitosterol
371	36	Lupeol
385	6	Phenol
388	6	2-Methyl-Dodecane-5-One
390	22	Dimethyl Azelate
393	22	Dimethyl Sebacate
398	6	6-Undecanol
400	20	Dimethyl Camphorate
402	20	Cedrol
405	6	Decanal
407	6	Nonanal
410	2	Guaiacol
412	6	6-Undecanone
420	24	Stigmasterol-Î’ -D-Glucoside
422	28	Astragaloside Viii
431	26	O-Cresol
433	4	2,3-Dicresol

438	26	Dihydropinosylvin
443	24	P-Cresol
447	18	M-Cresol
450	12	beta-Chamigrene
452	24	()-Cuparene
461	8	5-Hydroxycoumarin,Guaiacol
464	8	Isoeugenol
466	6	Astragaloside I
468	4	Astragaloside Ii
502	6	Glucuronic Acid
511	10	Ethyl-P-Methoxycinnamate
532	6	1,7-Dihydroxy-3,9-dimethoxy pterocarpene
595	2	Carvacrol Acetate
643	10	1,1,5-Trimethyl-2-Formyl-Cyclohexa-2,5-Diene-4-One
692	4	senkyunolide-D
705	14	Beta-Myrcene
707	14	Tetradecane
709	14	Beta-Elemene
711	14	1-Tridecene
714	14	Alloocimene
720	14	Camphene
722	14	1,2-Dimethylbenzene
726	14	Beta-Caryophyllene
728	14	1-Dodecene
779	6	Phyllanthin
974	8	2',4'-Dihydroxyacetophenone
985	12	senkyunolide-C
988	10	senkyunolide-E
999	4	9,10-dimethoxypterocarpan-3-O-β-D-glucoside
1096	2	Niacin,Nicotinic Acid
1131	2	Maruzen M,P-Ethylphenol
1202	2	Acetyl Astragaloside I
1206	2	Isoastragaloside I
1331	2	Scopoletin
1346	2	Vitamin B1
1452	2	Soyasaponin 1
1464	6	Isococculidine
1590	2	4-Octanone
1629	2	Hexanoic Acid
